# Social network structure is predictive of health and wellness

**DOI:** 10.1371/journal.pone.0217264

**Published:** 2019-06-06

**Authors:** Suwen Lin, Louis Faust, Pablo Robles-Granda, Tomasz Kajdanowicz, Nitesh V. Chawla

**Affiliations:** 1 Department of Computer Science and Engineering, University of Notre Dame, Notre Dame, IN, United States of America; 2 Interdisciplinary Center for Network Science and Applications, University of Notre Dame, Notre Dame, IN, United States of America; 3 Department of Computational Intelligence, Wroclaw University of Science and Technology, Wrocław, Poland; Cinvestav-Merida, MEXICO

## Abstract

Social networks influence health-related behavior, such as obesity and smoking. While researchers have studied social networks as a driver for diffusion of influences and behavior, it is less understood how the structure or topology of the network, in itself, impacts an individual’s health behavior and wellness state. In this paper, we investigate whether the structure or topology of a social network offers additional insight and predictability on an individual’s health and wellness. We develop a method called the Network-Driven health predictor (NetCARE) that leverages features representative of social network structure. Using a large longitudinal data set of students enrolled in the NetHealth study at the University of Notre Dame, we show that the NetCARE method improves the overall prediction performance over the baseline models—that use demographics and physical attributes—by 38%, 65%, 55%, and 54% for the wellness states—stress, happiness, positive attitude, and self-assessed health—considered in this paper.

## Introduction

Social networks play an important role in the diffusion of behavior, attitudes, tastes, and beliefs. Several studies have shown that such characteristics leverage the existing social connections and ties for diffusion. This phenomenon is demonstrative of the similarity or *homophily* between the nodes in the network (ego and alter, for example) and also of the social influences that affect people. Some examples of this diffusion process include: the spread mechanism of diverse health conditions over social networks—such as obesity [1] and smoking [2], the effect of social network on personal psychological traits—such as affection [3] and happiness [4], and the spread of health behavior through social networks [5]. People’s interactions through social networks or social media platforms have also been used to discover aspects of emotions experienced by individuals [6], mental illness [7, 8], and activity patterns [9]. Different social network types, such as friendship or non-friendship networks, can also provide insights about mental health in adults [10].

The network effect hypothesis suggests that similarities in lifestyle and health practice, including health behavior, moods, emotions, cultural norms, etc. [11, 12], among individuals is also a result of influence and diffusion within their network through their ties. In addition, the self-selection hypothesis suggests that ties among people are driven by similar pre-dispositions to attitudes or beliefs or behavior, so those factors might even be driving the formation of the tie [13, 14]. What is the inter-play between the network effect theory and self-selection hypothesis on its influence of individual’s health or wellness state? This paper considers wellness states to comprise of the attributes of stress, happiness, positive attitude, and health. To what end does the social network structure, in itself, influence the prevalence or influence of health / wellness states of individuals? What role do individual attributes such as demographics and health behavior play in the wellness state of individuals? What is more predictable of a wellness state—health behavior and demographic data or social network? Formally, the following are the two research questions that we answer in this paper that encompass our hypotheses and questions raised above.

### RQ1: Is social network structure indicative of health behavior? (Analysis)

**Fig 1:**
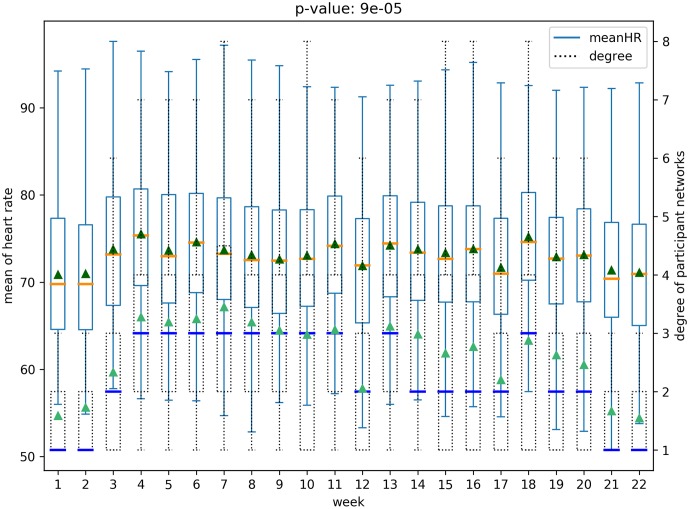
Main result for the relation between network structure and health behavior.

A social network structure can be measured by network properties such as node degree, clustering coefficients, and centrality. And we consider the health behavior as data captured from wearable devices—heart rate, daily steps, and activity states—and gender as the demographic data (the cohort is all similar age group of college going students). We analyze, quantitatively and qualitatively, the relationship between the social network structure and the aforementioned health information. An example of this relation is shown in Fig 1. This figure shows how the node degree on the network (shown in dashed box-plot) is related to the changes in the heart rate (shown with regular-lines). The figure also represents that these values seem positively correlated because, as time progresses, the mean and the median of node degree (shown as blue lines and as green triangles, respectively) increase or decrease when the mean or median of the heart rate (show as orange lines and dark green triangles, respectively) also increase or decrease, where the corresponding normalized cross correlation is 0.84 (*p* < .05). In the subsequent sections, we provide evidence that social network structure contains information that captures the change in statistics of health behavior.

### RQ2: How predictable are the wellness states from the incorporation of social network structure? (Prediction)

**Fig 2:**
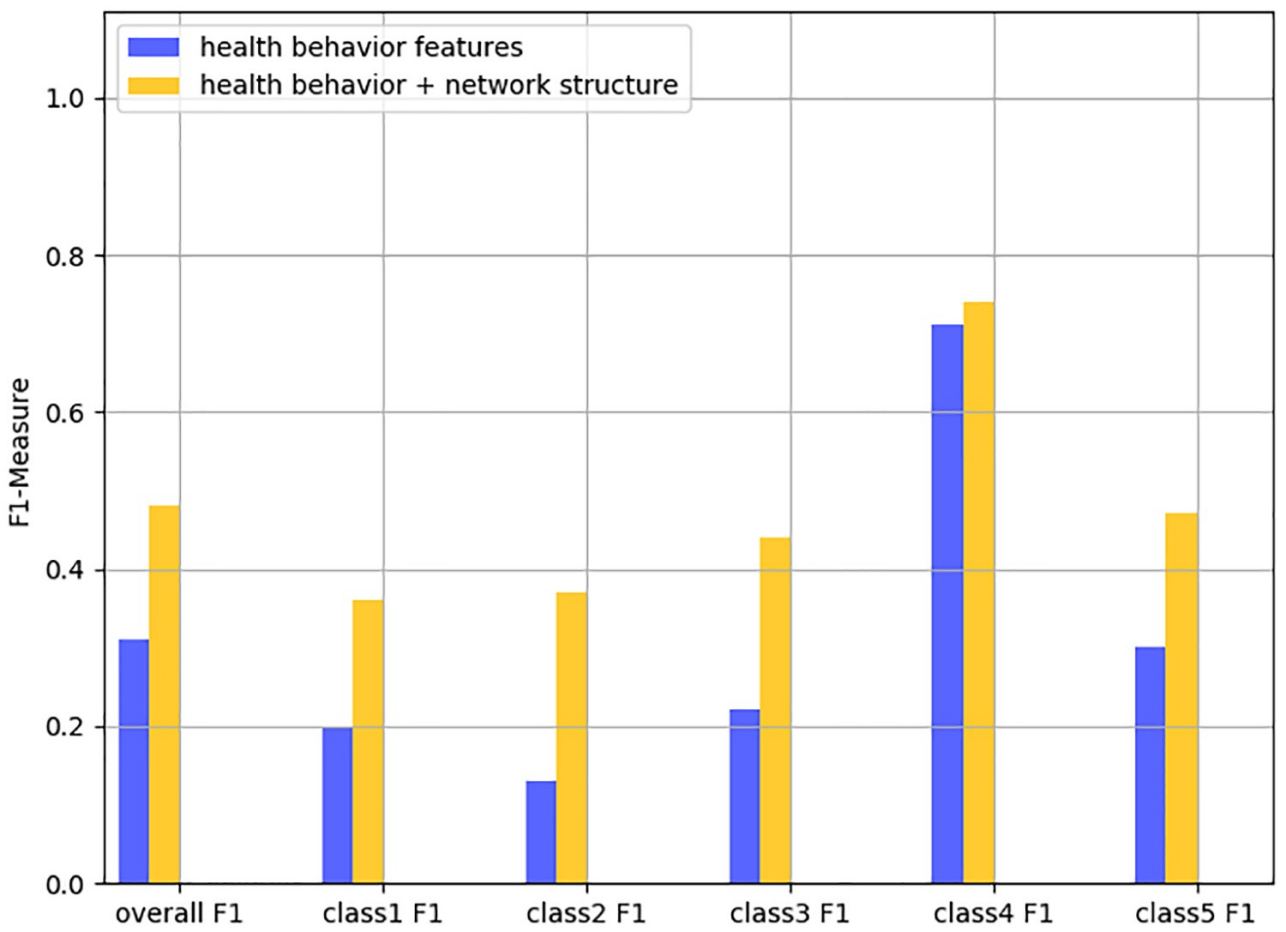
Main result for positive attitude prediction performance.

While previous research has shown that health behavior data captured from wearables is indicative of diseases or symptoms of diseases [15, 16], we incorporate the social network structural features in addition to health behavior data captured by wearables using a machine learning method (NetCARE) that predicts different states of health and wellness. We consider various wellness states such as stress, happiness, positive attitude, and self-assessed health indicators. Fig 2 summarizes the improvement of overall F1-Measure and within-class F1-Measure for positive attitude prediction by involving the network structural information. Clearly, the knowledge of social network structure (network effect) provides a significant improvement over using the data from the wearables and / or the individual’s demographics alone (self data).

## Methods

### Data

We use data from the NetHealth study [17], an ongoing project at the University of Notre Dame, collecting survey, phone and Fitbit data from an initial cohort of 698 first-year students who were enrolled in the Fall of 2015. All procedures were fully approved by the University of Notre Dame Institutional Review Board before distribution and performed in accordance with the relevant guidelines and regulations. All study participants provided informed consent and acknowledged all of the study goals, procedures, and data privacy, prior to any data collection.

**Fig 3:**
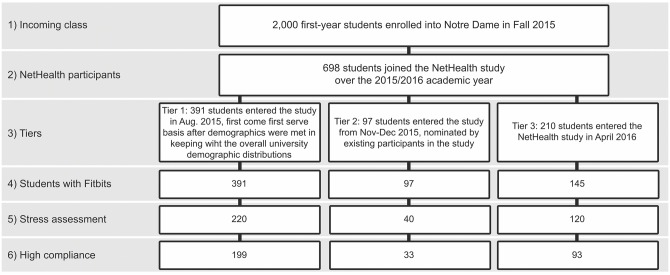
Consort diagram of NetHealth recruitment and students selected for this analysis.

An outline of the recruitment process and student sample numbers are provided in Fig 3. Participants were provided with a Fitbit Charge HR and had an app installed on their phone, which was leveraged to build the social network on the basis of communication patterns (call, message). They were also required to complete an entrance survey before arriving on campus and follow-up surveys after each semester. Surveys contain a battery of questions regarding individual demographics and self-reported mental and physical wellness assessments. It should be noted that the survey questions are different for each semester and not all students took part in all the surveys.

We consider the following three data sources from NetHealth:

1. *Health Behavior and Demographic Data*. The health behavior data, obtained from Fitbit devices, and includes health-related behavioral variables such as heart rate, step and activity states. Besides the minute-by-minute raw heart rate and step data, Fitbit also separates and tracks four activity states per minute based on METS, a weight-agnostic measure of activity, that represent sedentary, lightly active, fairly active, and very active activity states [18]. In addition, we also consider the gender of the participants in our analysis (the only demographic feature).
2. *Social Network Data*. The students’ social networks were constructed using their communication activities including texts and phone calls captured through an App installed on their phone for the study. This App can automatically gather the time, source and destination of their communication activities. As for phone calls, the App can also record the duration of the call and whether the call was answered.
3. *Wellness State*. These data are from surveys answered by participants each academic semester. Due to the different survey questions across semesters, we cannot jointly analyze all the surveys. For that reason, we selected the survey taken in Fall 2016, which contains questions about wellness states—stress, happiness, positive attitude and self-assessed health—and covers most of our participants (380 subjects). Accordingly, we considered contemporary data from fitness trackers and social interaction from August 2016 to December 2016. We excluded 47 participants for missing Fitbit or social network data. As a result, our data covers 325 participants.

Table 1 provides a demographic overview of our sample from the NetHealth study (not all participants report gender and race). Table 2 presents the four different wellness attributes we examine in this study, stratified by different levels based on their respective Likert scales. For *Stress*, lower levels indicate less stress and higher levels indicate more stress. Regarding *Happiness*, *Positive Attitude* and *Health*, lower levels indicate more negative outlooks such as no happiness or poor health and higher levels indicate more positive outlooks.

**Table 1:** Summary of demographics in data samples.

demographic		# Data Points
*gender*	male	146 (45%)
	female	179 (55%)
*race*	white	227 (70%)
	latino	36 (11%)
	asian	29 (9%)
	black	18 (6%)
	foreign	14 (4%)
*age*	17	36 (11%)
	18	182 (56%)
	19	11 (3%)

The total number of corresponding subjects are 325.

**Table 2:** Summary of wellness-related survey data in the NetHealth study.

Wellness State	Level 1	Level 2	Level 3	Level 4	Level 5
	#P(#M,#F)	#P(#M,#F)	#P(#M,#F)	#P(#M,#F)	#P(#M,#F)
**Stress**	14	95	134	82	–
You felt nervous and stressed	(12,2)	(58,37)	(52,82)	(24,58)	
**Happiness**	40	84	144	57	–
You were happy	(14,26)	(38,46)	(64,80)	(30,27)	
**Positive Attitude**	2	23	65	160	75
You took a positive attitude	(0,2)	(8,15)	(28,37)	(72,88)	(38,37)
**Health**	7	56	200	62	–
Health Rating	(4,3)	(18,38)	(95,105)	(29,33)	

The total selected participants are 325. (Notation: #P, #M and #F are the number of all participants, that of male participants and that of female participants in the corresponding level, respectively).

### Data preprocessing

There are two steps for data preprocessing. First, to ensure there is no bias between the students with different levels of sparsity in daily Fitbit data, we eliminated samples with less than 80% daily wear time (19 out of 24 hours) in our analysis as this threshold has been shown to provide reasonable estimates of students activity [17]. Second, we aggregated the data from August 2016 to December 2016 into weekly time-points, where each data point includes the given week’s Fitbit data, social data and corresponding survey data from the Fall 2016 survey. The data was aggregated in this manner to better fit the streaming Fitbit and social network data to the single survey outcome.

### Feature extraction

We extracted several features, detailed in the following subsections, from these data streams to build an appropriate feature vector for the learning algorithms.

#### Gender information

The World Health Organization has recognized gender differences in stress-related syndromes [19]. For example, females have much higher incidence rates of stress than males. Based on this insight, we extracted the gender information from the survey data to use as an additional feature in the feature vector. Table 2 shows the population distributions for the different levels of survey variables for males and females. Specially, consider the case of stress, happiness, and health, males mainly fall into level 2 and 3, and most of the females fall into level 3. We use gender as an independent variable (predictor) in our analysis.

#### Health behavior data

We categorize the physical attributes captured from Fitbit (heart rate, steps, and activity states) as health behavior data. This data is segmented into the weekly intervals discussed in the previous section. Then, summary statistics of mean and variance (or standard deviation) are computed on these temporal segments.

**Heart Rate**. We computed the mean and variance for the heart rate over each week for each participant. We also applied ANOVA tests to examine the heart rate differences among different stress levels, happiness levels, health levels, and positive attitude levels. Results showed significant differences of heart rates for different stress levels (*p* < .001), happiness levels (*p* < .001), health levels (*p* < .001), and positive attitude levels (*p* < .001).

**Steps**. The raw data for steps are also recorded minute by minute, but it is more likely to be zero for most of the minutes in one week due to the nature of walking. Thus, we first transformed the raw minutely step data into the sum of steps each day. Then, we computed the mean and standard deviation of these daily steps for each week and each person as features.

**Activity State**. Fitbit tracks the users’ activities and records their corresponding pre-defined states every minute. There are 4 possible states: sedentary, lightly active, fairly active and very active. So, the sum of minutes in each state on each day are computed first, then mean and standard deviation of these daily summations for each state within each week are computed.

#### Social network data

Social networks were constructed from the communication patterns of phone calls and text messages. To avoid spurious connections (such as spam), we eliminated communication edges that had a frequency of fewer than three times within a 5-month period. The NetHealth study collected communication data not only from within all the participants but also between participants and people from outside of the study as well. As a result, we had two types of social networks: one that includes all the data (*whole network*) and the one that only includes communication patterns of the participants within the study (*participant network*). The *participant network* only includes friends or classmates since all the participants are undergraduate students with the same class-standing or year in the same university. We can regard the *participant network* as a *friend network*, which is one of the five types of social networks that can affect health [10]. However, the *whole network* contains more complete information of each ego structure, so we studied both the *whole network* and *participant network* in our analysis.

As we mentioned before, each time step in our social network analysis consists of one week. The social networks are undirected and unweighted representations of communication patterns for each week. We then derive several features that are representative of the social network structure, including network degree [20], number of triangles, clustering coefficient [21], betweenness centrality [22], and closeness centrality [23] for each person in the network.

### Analysis framework

#### Health behavior relationship analysis

To answer RQ1, we investigate if there is a relationship between social network structure and health behavior and whether the social network structure properties are predictive of the health behavior. Specifically, we examine the relationship between social network topological properties including degree, number of triangles, clustering coefficient, closeness centrality and betweenness centrality for each node (individual) in the *participant network* and in the *whole network* and health behavioral variables including heart rates, steps and activity states.

We visualize all the 22 weeks using box plots to show the relationship between network structural variables and health behavioral statistics in a qualitative way. On the other hand, we use cross correlation coefficients [24] to quantitatively capture the correlation between each of the behavioral variables and each of the network structural variables over all participants. It should be noted that the links from physical and behavioral variables to social network variables can vary across individuals. Thus, we further compute the correlation for each individual, and then sum up the total number of coefficients showing a value greater than 0.5.

#### Wellness state prediction

**Fig 4:**
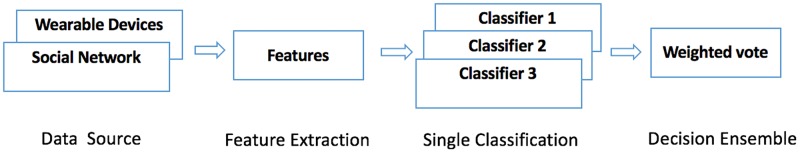
A network-driven prediction method, NetCARE.

To answer RQ2 we propose NetCARE, a network-driven prediction method, to make full use of social network structure features in health prediction problems. Fig 4 shows the schema of NetCARE. The method incorporates social network structure, wearable data, and demographic data as independent variables of a machine learning model. This algorithmic architecture allows us to select network features and add other data sources as needed. It also ensures the flexibility to modify, extend, or add classifiers.

As mentioned earlier, we predict four wellness states: stress, happiness, positive attitude and self-assessed health. Tables 1 and 2 present the diversity of our participants across race, gender and levels of wellness states. Let us use stress prediction as an example to explain this architecture in detail. The stress prediction problem is formulated as a 4-categories classification problem based on the different stress levels in Table 2. The features were extracted from health behavior and network structure, and the class categories are the levels of stress. We then employed five popular classifiers for the problem: K-Nearest Neighbors (KNN), Classification and Regression Trees (CART), Support Vector Machines (SVM), Logistic Regression (LR) and Random Forests (RF). The data was divided into 75% for training and 25% for testing. We used 5-fold cross-validation on the training data to find the hyper-parameters of the algorithms, and used grid search to find the combination of those parameters that achieved the best performance. We then consider the averaged F1-Measures for all levels and those within each level as a metric of performance. Specifically, we tuned the number of neighbors for KNN and the leaf size for CART. For SVM, we conducted experiments over the three different kernels: polynomial, linear and radial basis function (RBF) kernels, where various degrees of polynomial kernels were also taken into consideration. For LR, we searched on different values for regularization coefficients and the learning rate of the optimization algorithm. For RF, we conducted experiments with different numbers of trees from 10 to 100 at increments of 5. We use 35 trees for the results reported in this document.

Furthermore, we applied an ensemble method with a weighted voting [25] scheme to improve the overall predictabive performance. We chose the three single classifiers with highest 5-fold cross-validation accuracy scores on the training data as base classifiers to use in the ensemble method. Specifically, they were SVM, KNN and RF. Let *p*_*ij*_ = *w*_*ij*_ represent the probability of classifier *i* classifying the input instance *x* as class *j*. The ensemble rule for combining the outputs of different base classifiers to get the the final prediction *y*_*vote*_ can be formulated as Eq (1). The optimal weights, $\left\{w_{ij}^{optimal}\right\}$, were selected using cross-validation on the training from all possible combination of weight *w*_*ij*_ from 0 to 1 with interval 0.1.

$$\begin{array}{c} \begin{array}{cc} & y_{vote}\left(\left\{w_{ij}\right\}\right)=\underset{j}{\text{argmax}}\sum_{i=1}^{3}w_{ij}p_{ij}\\ & s.t.\;\sum_{j=1}^{4}w_{ij}=1,j\in \left\{1,2,3,4\right\},\;\text{and}\;i\in \left\{1,2,3\right\} \end{array} \end{array}$$ (1)

Our experiments also include a benchmark method for comparison, where the prediction of the wellness states were randomly generated among all the potential levels with equal probabilities.

## Results

Based on the discussed methods and framework, we performed two sets of experiments. First, we evaluated the interactions among the variables associated with social network structure and those related to health behavior. The objective of this analysis was to validate whether their interactions were meaningful. Second, we used our framework to predict various wellness states. We compared the performance of our framework with two baselines. One of them applies our framework to either health-behavior data or network features in isolation and the other one comes from the random generation. The objective was to verify our hypothesis that combining network effects and self-similarity would lead to better predictions.

### Health behavior relationship analysis

**Fig 5:**
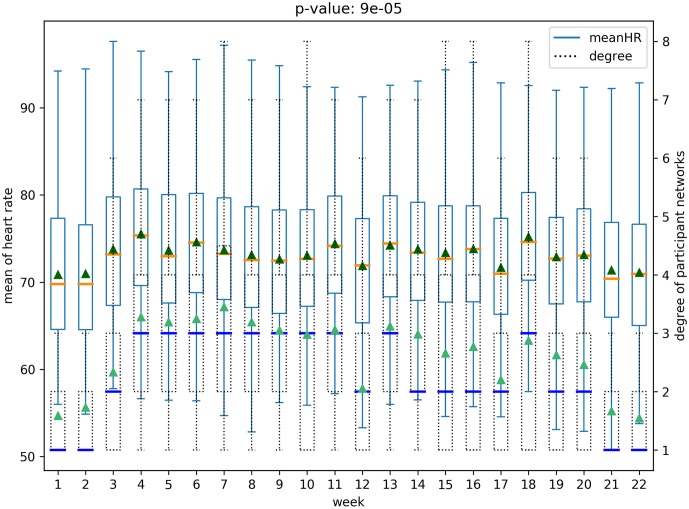
Relation between heart rate and degree of *participant network*.

**Fig 6:**
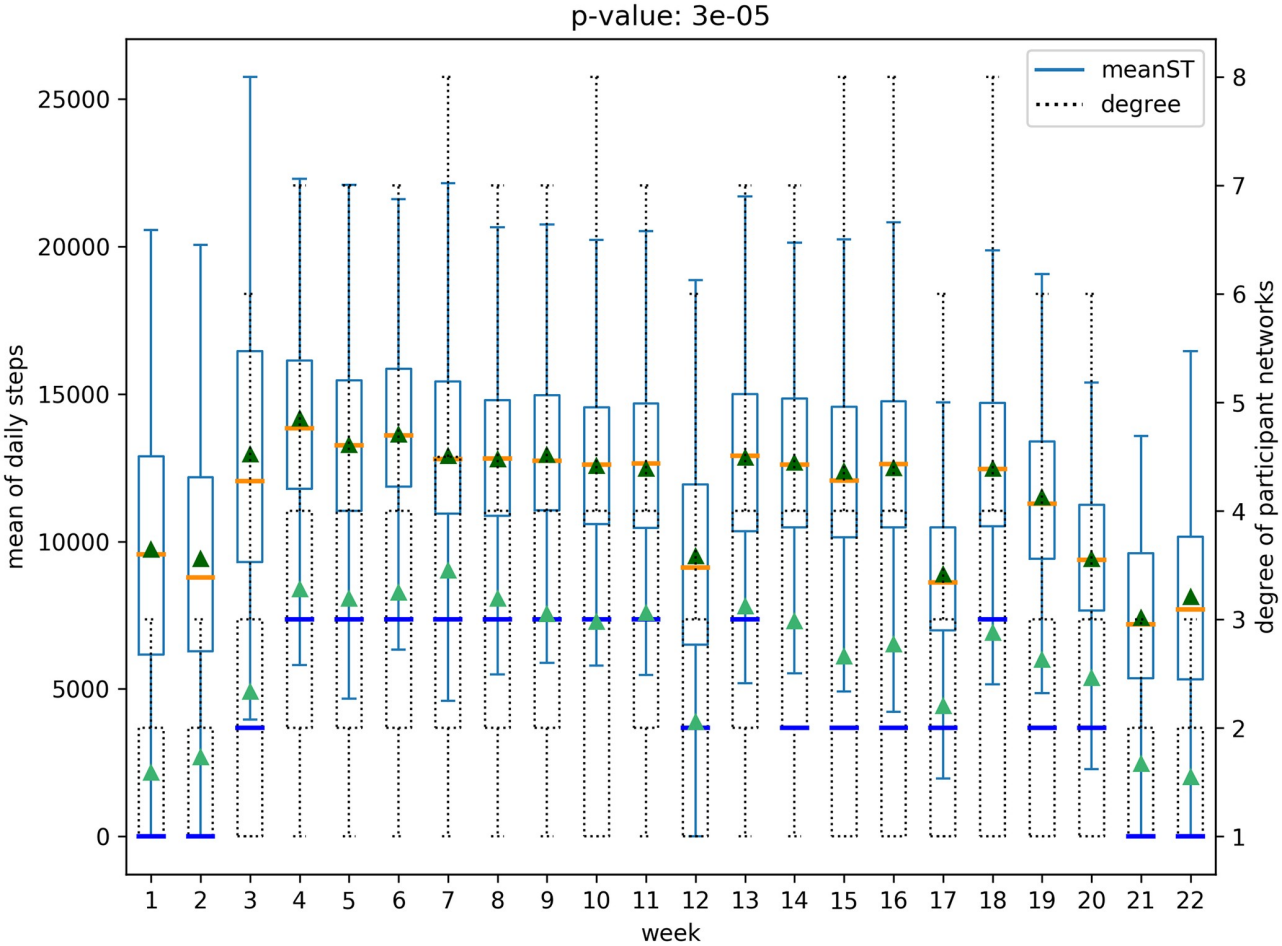
Relation between daily steps and degree of *participant network*.

**Fig 7:**
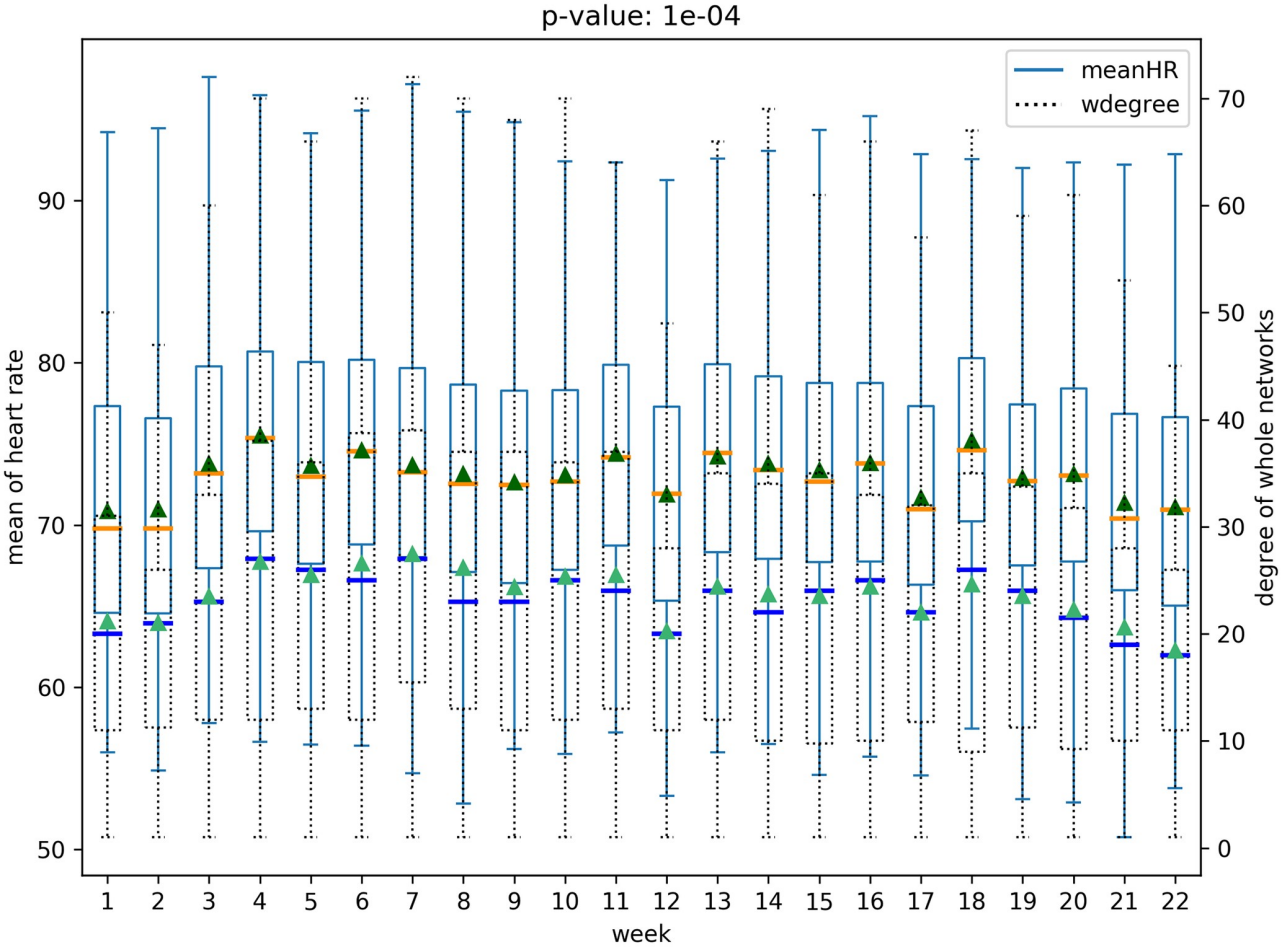
Relation between heart rate and degree of *whole network*.

**Fig 8:**
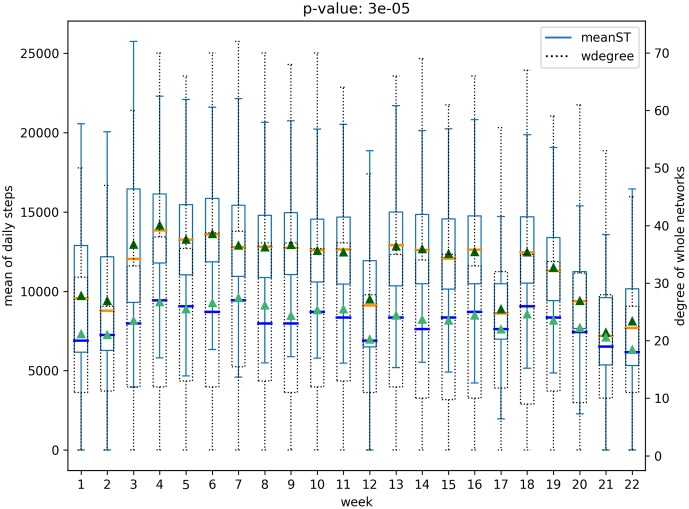
Relation between daily steps and degree of *whole network*.

We analyze all possible pairs of five structural features of social networks in both the networks—(*whole network* and *participant network*)—and six physical and behavioral features with box plots, resulting in sixty box plots. Each boxplot includes the distribution of a physical-behavioral feature and a social network structure feature for all the participants over the 22 weeks period. Note that health-behavioral features for each week were extracted as mean values from the raw data. For example, Fig 5 presents the distribution of average heart rates for all participants and the node degree distribution over the 22 weeks of the *participant network*. Figs 5 and 6 represents the relationships for the *participant network* structures, while the relationships for the *whole network* are presented in Figs 7 and 8. Note that the remaining box plots can be found in the supplemental material S1 Appendix.

As shown in Figs 5, 6, 7 and 8, the median and the mean of health behavior data for each week (dark orange lines and dark green triangles in the figures, respectively) change over time and the median and mean of network properties (dark blue lines and sea green triangles in the figures, respectively) follow a similar pattern over time.

We performed tests to verify whether there was a statistically significant difference between the distribution of health behavior features and the network structure features across high and low-value ranges. Specifically, using t-tests we tested for whether the feature values representative of behavioral data varied in the strength of the relationship with the network data ranges. For example, consider the relationship between daily steps and network degree. The derived p-value of 0.0003 shows that there is a significant difference between the daily steps in conjunction with higher network degree versus daily steps in conjunction with lower network degree. The corresponding p-value is shown in Fig 5. After correcting for multiple tests [26], our results show that social network properties have significant relationships 43 out of 60 times, supporting the hypothesis that social networks are indicative of changes in health behavior.

Further, we used cross correlation coefficients [24] to quantify the extent to which the network structure features can reflect the information flow of health behavior. After calculating the means of each feature from health behavior and network structure for every week, we computed the coefficients of the means of health behavior features and the means of structure features (sixty pairs). The results showed 43 of 60 pairs with a absolute correlation coefficient that is no less than 0.5 and 28 of the 60 pairs with absolute correlation no less than 0.7. Table 3 shows the results from all the pairs of behavioral features and network structure features.

**Table 3:** Normalized cross correlation coefficients of each pair of health behavior feature averages and social network structure feature averages.

Network Structure	heart rate	steps	sedentary	lightly active	fairly active	very active
Degree in *participant network*	0.84*	0.89*	-0.44*	-0.014	0.49*	0.87*
Number of triangles in *participant network*	0.74*	0.83*	-0.61*	0.24	0.68*	0.79*
Clustering Coefficient in *participant network*	0.65*	0.75*	-0.51*	0.15	0.59*	0.66*
Betweenness Centrality in *participant network*	0.78*	0.68*	-0.19	-0.20	0.20	0.72*
Closeness Centrality in *participant network*	0.83*	0.85*	-0.32	-0.14	0.35	0.86*
Degree in *whole network*	0.81*	0.90*	-0.57*	0.15	0.62*	0.88*
Number of triangles in *whole network*	0.79*	0.89*	-0.62*	0.23	0.69*	0.85*
Clustering Coefficient in *whole network*	0.83*	0.79*	-0.32	-0.12	0.35	0.79*
Betweenness Centrality in *whole network*	-0.76*	-0.85*	0.65*	-0.28	-0.71*	-0.79*
Closeness Centrality in *whole network*	0.75*	0.78*	-0.59*	0.24	0.65*	0.71*

The correlation values with significant values (*p* < .05) are marked by asterisks.

The correlation from network-structure features to the very active state is generally stronger by about 0.3 on average than that from structure to sedentary, fairly active or lightly active states. We noticed none of the structure features have a strong relation to the data of the lightly active state. After excluding results of the lightly active state, we found that the number of triangles in *participant network* and *whole network* can be a good indicator of the change of other health behavior data. Also, except lightly active, each behavioral feature could be related to at least one of the structural features with absolute coefficients no lower than 0.7. Especially, the correlation coefficient between degree in the *whole network* and steps is almost 0.9. We did the same experiments on the medians of each feature for every week. We found 37 of 60 pairs had correlations no less than 0.5. Comparing the results from metrics in *participant network* with those from metrics in *whole network* in the table, we observed major differences between the two networks. For example, the coefficient between the fairly active state data and Closeness Centrality in the *participant network* is almost half of coefficient between fairly active data and Closeness Centrality in the *whole network*, while the coefficient between fairly active data and Clustering Coefficients in the *participant network* almost doubles the coefficient between fairly active data and Clustering Coefficients in the *whole network*. This finding suggests that there are different effects in the two network types and it is necessary to include both networks in our analysis. In summary, Table 3 show that the network structure seems to capture the changes of health behavior—although in a lesser extent with respect to the lightly active state.

We also evaluated variable interactions for each participant in the dataset. This was done to evaluate changes in health behavior per individual. Each analyzed sample point is the average of the behavior over one week per person. We counted the total number of persons with more than 0.5 on absolute cross correlation coefficients to show the extent to which the structural features can capture the changes in health behavior. Table 4 lists the numbers of participants with medium to strong correlation for each pair of health behavioral features and network features. In the table, each health behavior feature can be related to one of structural features for both *whole network* and *participant network* for at least 20% of participants. The table shows that Closeness Centrality in either the *whole network* or *participant network* capture a relationship with steps for over 42% of the samples. These results imply an underlying relationship between features representative of the social network structure and the health behavior.

**Table 4:** Number of persons whose health behavior have medium to strong correlation with social network structure.

Network structure	heart rate	steps	sedentary	lightly active	fairly active	very active
Degree in *participant network*	52	86	43	39	42	47
Number of triangles in *participant network*	31	38	29	24	37	26
Clustering Coefficient in *participant network*	28	37	27	24	35	27
Betweenness Centrality in *participant network*	38	38	34	35	32	34
Closeness Centrality in *participant network*	99	145	81	79	82	98
Degree in *whole network*	78	94	81	58	70	68
Number of triangles in *whole network*	63	100	69	43	62	60
Clustering Coefficient in *whole network*	47	66	46	37	51	39
Betweenness Centrality in *whole network*	50	52	57	54	53	49
Closeness Centrality in *whole network*	91	137	98	83	94	80

Table 5 summarizes the fraction of participants with medium to strong correlations with respect to health behavior features and at least one graph structure feature from *participant network*, *whole network* or both networks, respectively. Particularly, the percentages are the fraction of persons whose health behavior data has no less than 0.5 cross correlation coefficients with any of the network structure features. For example, the person whose any one of 5 features from *participant network* is related to steps data with coefficients no less than 0.5, is counted. According to the first and second columns in Table 5, the three metrics from each kind of social networks can closely capture the changes of some health behavior for about 50% of the participants. Specifically, 74% of the participants have higher correlations between steps and one aspect of structural features. The last column in Table 5 shows that both types of social networks maintain information of the time-varying heart rate averages, step averages, and averaged minutes in each activity states, for over 50% participants. Additionally, the fraction of persons with correlation coefficients no less than 0.7 between the number of steps and the structural features in *whole network* or in *participant network* is 40%, and between the mean of heart rates and the structural features is 26%. These results imply the *whole network* contains more sufficient information about health behavior than the *participant network*, but both of them are essential pieces, given the increase of numbers in the last column.

**Table 5:** Summary of subjects with medium to strong correlation to the social network structure.

health-related data	*participant network* (%)	*whole network* (%)	both network (%)
heart rate	133 (41)	157 (48)	**193 (59)**
steps	**182 (56)**	**204 (63)**	**239 (74)**
sedentary	125 (38)	**166 (51)**	**202 (62)**
lightly active	132 (41)	145 (45)	**199 (61)**
fairly active	122 (38)	**164 (50)**	**194 (60)**
very active	133 (41)	143 (44)	**186 (57)**

Percentages are the fraction of persons whose health behavior data has no less than 0.5 cross correlation coefficients with any of the network structure features, where total number of persons in the data is 325.

In summary, these experiments verify the interactions among network-structure variables and health-behavioral variables. Specifically: **1)** We demonstrated that the network structures can qualitatively capture the changes of behavioral variables. **2)** We conducted t-tests to check if higher values of health-behavior variables corresponded to higher values of structural variables and are different from lower values of both types of variables. Our results showed 43 out of 60 with significant differences after multiple-test corrections. **3)** We used normalized cross correlation coefficients to describe the role of network structures in statistics. The results showed 43 of 60 pairs of behavior features and structural features with a correlation coefficient that is no less than 0.5 and about half of the pairs with a coefficient no less than 0.7. **4)** We analyzed the variable interactions of structure and physical features at the individual level and found that up to 145 out of 325 participants showed a high correlation between their closeness centrality of networks and steps, and up to 74% of the participants showed the similar relation between the aggregated network features and steps.

### Predicting wellness state

After implementing the five single classifiers, we chose SVM, KNN, and RF, to create our ensemble learning model. The first five rows of Table 6 show the performance of our ensemble classifiers for stress prediction.

**Table 6:** Prediction results for happiness, positive attitude and self-assessed health.

**Stress Prediction**	F1	Level1	Level2	Level3	Level4	
random generation baseline	0.21	0.04	0.23	0.32	0.24	
gender + health behavior data	0.42	0.18	0.53	0.64	0.34	
social network structure	0.34	0.05	0.43	**0.63**	0.26	
gender + health behavior data + social network	0.58	0.46	0.63	0.70	0.55	
improvement	38%	**155%**	19%	9%	62%	
**Happiness Prediction**	F1	Level1	Level2	Level3	Level4	
random generation baseline	0.24	0.16	0.26	0.31	0.24	
gender + health behavior data	0.31	0.06	0.31	0.62	0.24	
social network structure	0.21	0.00	0.2	0.60	0.02	
gender + health behavior data + social network	0.51	0.43	0.52	0.67	0.44	
improvement	65%	**617%**	68%	8%	83%	
**Positive Attitude Prediction**	F1	Level1	Level2	Level3	Level4	Level5
random generation baseline	0.17	0.03	0.11	0.19	0.31	0.20
gender + health behavior data	0.31	0.20	0.13	0.22	0.71	0.30
social network structure	**0.40**	**0.70**	0.08	0.23	0.70	0.25
gender + health behavior data + social network	0.48	0.36	0.37	0.44	0.74	0.47
improvement	55%	80%	**185%**	**100%**	4%	57%
**Self-assessed Health Prediction**	F1	Level1	Level2	Level3	Level4	
random generation baseline	0.19	0.01	0.19	0.34	0.20	
gender + health behavior data	0.35	0.29	0.13	0.77	0.20	
social network structure	0.21	0.00	0.05	0.77	0.00	
gender + health behavior data + social network	0.54	0.6	0.39	0.79	0.37	
improvement	54%	**107%**	**200%**	3%	85%	

The improvement in the table is to compare the performances from the health behavior and gender features with those from integration of health behavior, gender and network features.

We report the F-score for all stress levels and each level. The table shows that social network variables alone are comparable or even a little better to health behavior data for overall F-score and stress level 3, while others are worse. Thus, we suspect social network structure contains information about stress from a complementary perspective compared to that of health-behavior variables, i.e. there seems to exist an underlying relationship between social network structure and stress state. The table also shows that joining features from social networks and health behavior improve predictions as evaluated by the F1-score improvement on both the combined performance and the individual performance per stress level. The most noticeable improvement corresponds to stress level 1.

Additionally, we perform the same analysis for other wellness states. In particular, we assess the effect of combining social network structure variables and health-behavior variables to predict wellness states of happiness, positive attitude and self-assessed health (Table 2). These results are also shown in Table 6. As in the case of stress, the table supports that using social network structure can improve prediction performance for these 3 health and wellness variables. Table 6, shows that our NetCARE provides improvements of: 1) 65% and up to 617% on the overall F1-Measure and the within class F1-Measure of *happiness*, respectively; 2) 55% and up to 185% on the overall F1-Measure and the within class F1-Measure of *positive attitude*, respectively; and 3) 54% and up to 200% on the overall F1-Measure and the within class F1-Measure of *self-assessed health*, respectively. These results provide evidence that not only structural features could be helpful in applications of wellness state prediction and health perceptions.

## Discussion

The main contributions of this paper can be summarized as follows:

- We discovered that social network structure is correlated with health behavior data obtained from wearables and can capture the trends. This relationship between social network structure and health behavior is statistically significant.
- We demonstrated that social network structure is highly predictive of wellness states. This result is of importance as just relying on data derived from wearables and demographics does not express a complete picture about an individual, and one’s social network is an important element to understanding and predicting health and wellness.

Social network analysis has been used for health-related problems including mental health [4, 6], physical well-beings [1, 2], and illness [8, 27]. Most of the work has largely focused on social networks as a diffusion mechanism of health [1–5] or emotions [6–9]. This paper provides a novel perspective on the value of social network structure in not only understanding our health behavior but also in predicting the wellness states, above and beyond what the data from wearables or demographic tells us. Clearly, social networks are an important piece of the puzzle about our health and wellness. We showed that by including features derived from social networks, accuracy increases significantly and at times using only social network features adds more predictability. Specifically, we find that happiness and positive attitude have the most significant jump when using social network structure features in addition to health behavior and demographic data. This clearly demonstrates that it is the tight coupling of an ego’s social and health behavior that result in improved understanding and predictability of the ego’s wellness state.

There are additional insights that might also be gleaned by our study. Consider the correlation among structural variables and health behavior variables (see, e.g. Table 3). We observe a moderate to strong correlation between clustering coefficient and heart rate, steps, and high activity states which may capture participation in campus sports. These activities provide participating students with ample amounts of physical activity and tightly knit social groups, factors which have been previously shown to be associated with mental health [28, 29]. Further, it seems that it is easier for social network structure to capture the activity states when a person is either in an inactive state or at least fairly active, than if the person is lightly active. It could be indicative of the relationship between activity and gregariousness or extraversion of an individual. Also, as lightly active minutes include walking, the location of dorms, classes and other necessary destinations involved in a students daily routine may contribute significant noise to this level of activity. A future research direction is looking at more granular data and time windows to understand the immediacy of communication patterns with respect to activity states.

## Supporting information

S1 Appendix Supplemental materials. The remaining boxplots of Health Behavior Relationship Analysis.
(DOCX)
