# Supplementary material for: Social network structure is predictive of health and wellness
Source: PLoS One. 2019 Jun 6;14(6):e0217264. doi: 10.1371/journal.pone.0217264 (PMC6553705; doi:10.1371/journal.pone.0217264)
Supplement: S1 Appendix — The remaining boxplots of Health Behavior Relationship Analysis. (DOCX) [file pone.0217264.s001.docx]

**Supplemental Material**

In this material, we present the remaining box plot figures mentioned in our main text. Each one of them represents the box plot of health behavior features and that of network structure features. And we can clearly observe the median and the mean of health behavior data for each week (dark orange lines and dark green triangles in the figures, respectively) are changing over time, and interestingly, the median and mean of network properties (dark blue lines and sea green triangles in the figures, respectively) can almost catch up with those changes. In each plot, the p value is the result of the correspond t-test, which is between those means from heart rate corresponding to 20% highest network degree and those means from heart rate corresponding to 20% lowest network degree.


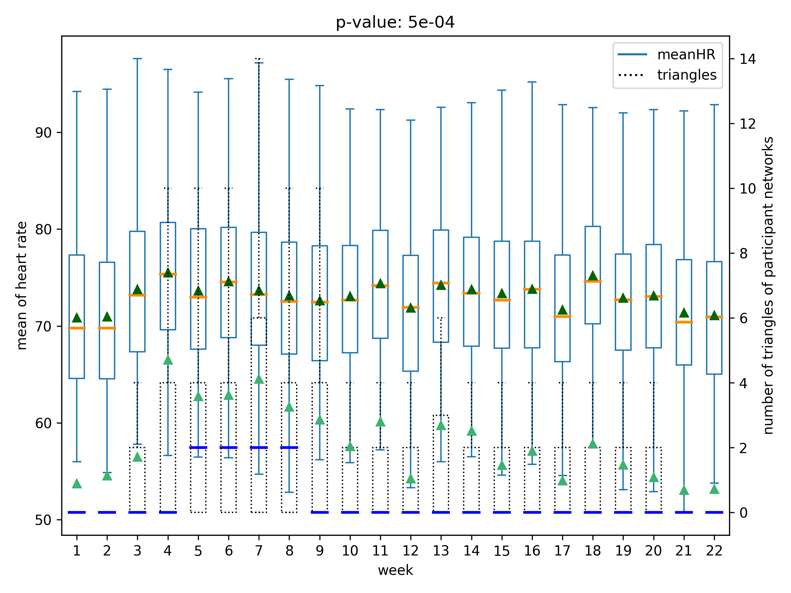


Figure 1 box plot of mean of heart rate and number of triangles of participant network


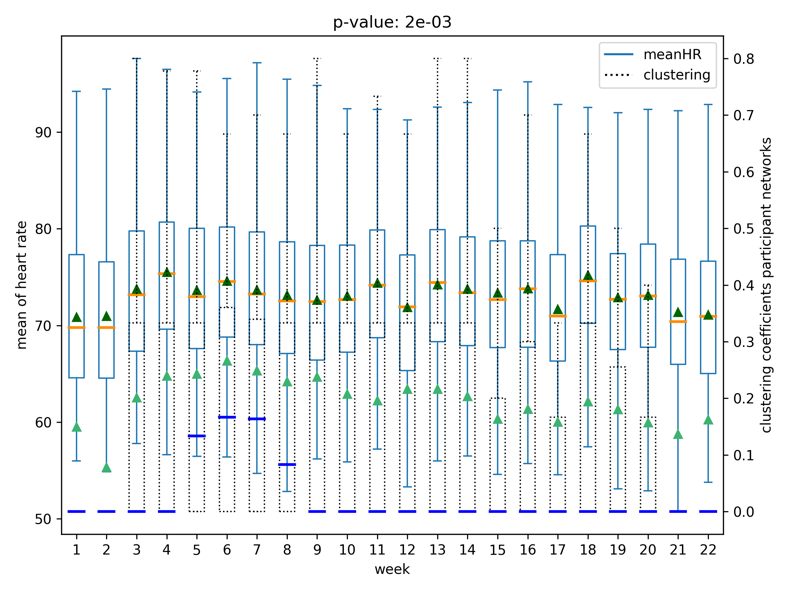


Figure 2 box plot of mean of heart rate and clustering coefficient of participant network


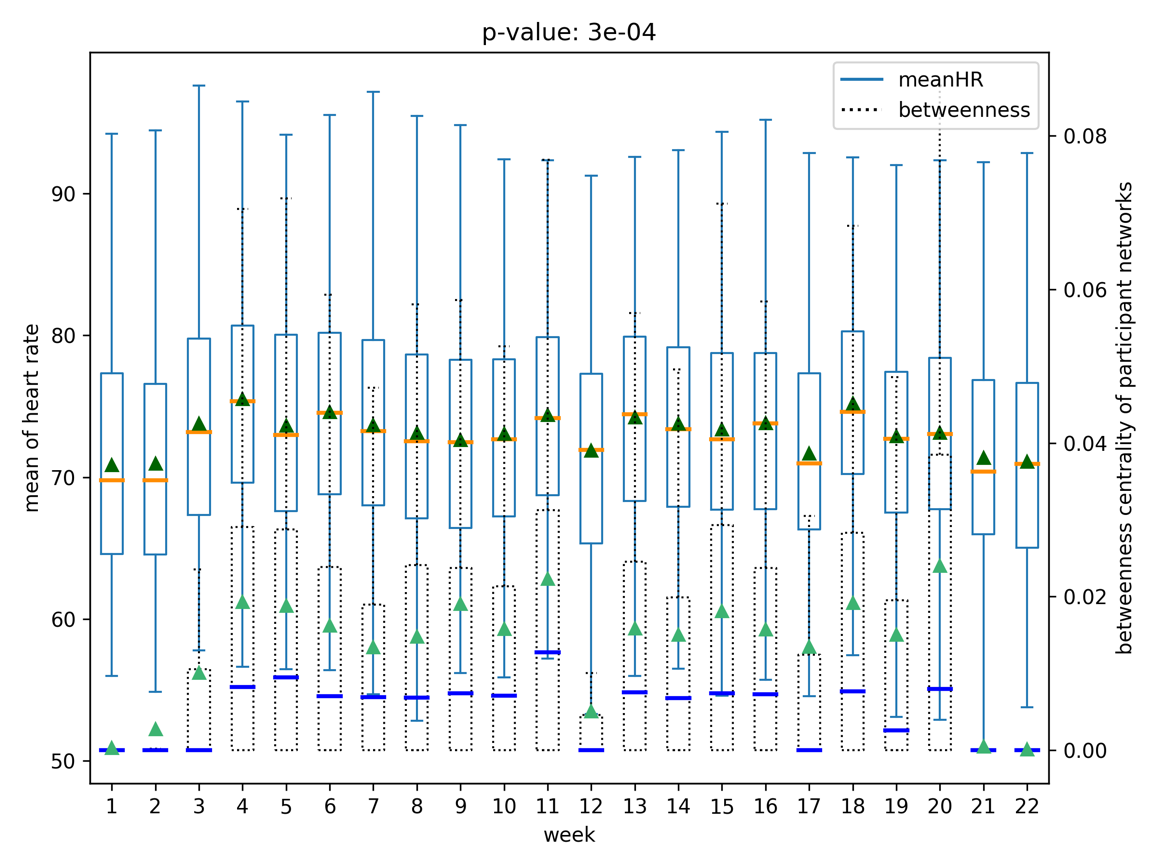


Figure 3 box plot of mean of heart rate and betweenness centrality of participant network


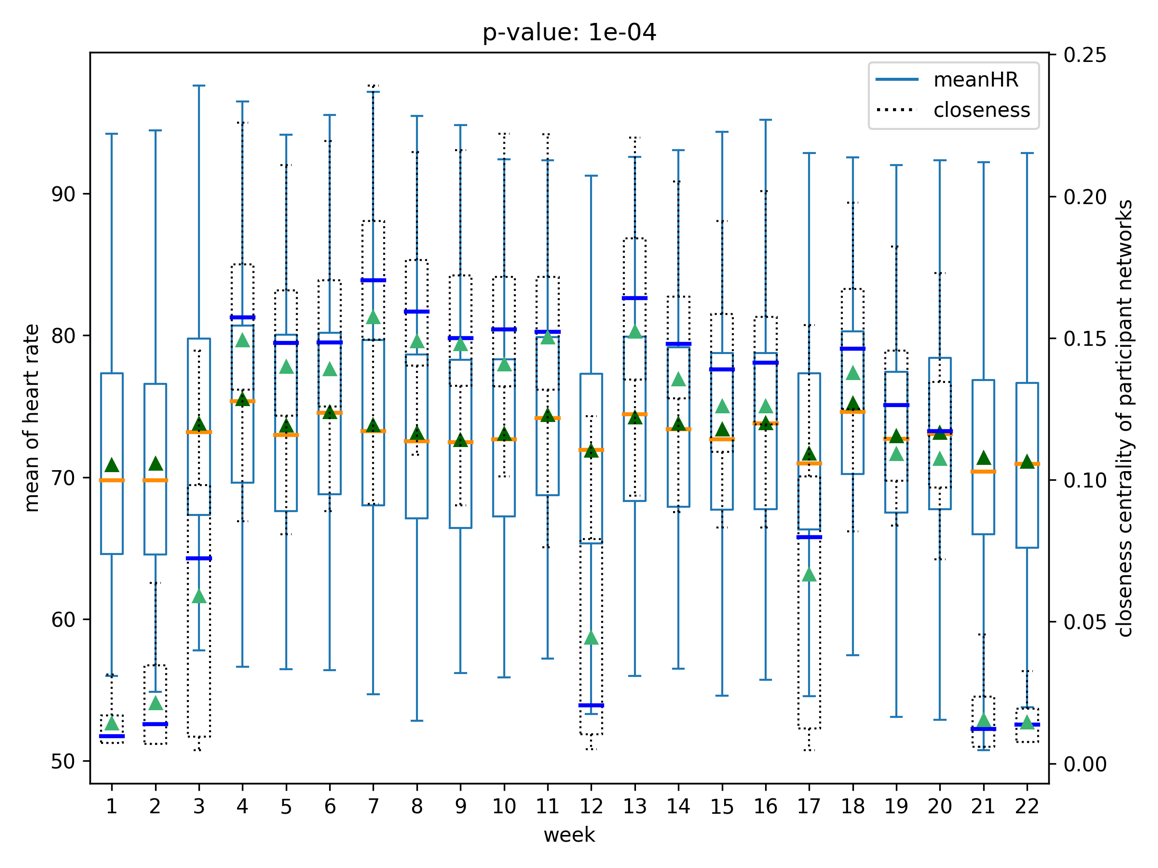


Figure 4 box plot of mean of heart rate and closeness centrality of participant network


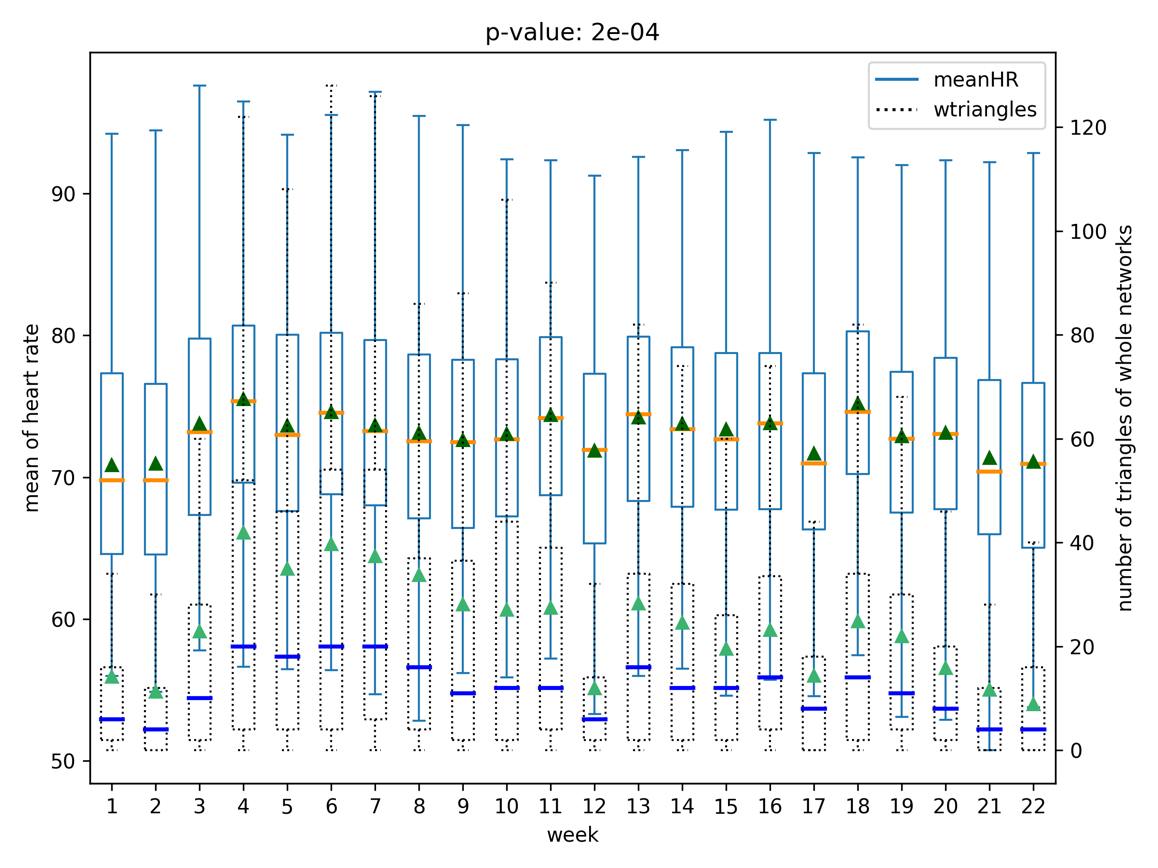


Figure 5 box plot of mean of heart rate and number of triangles of whole network


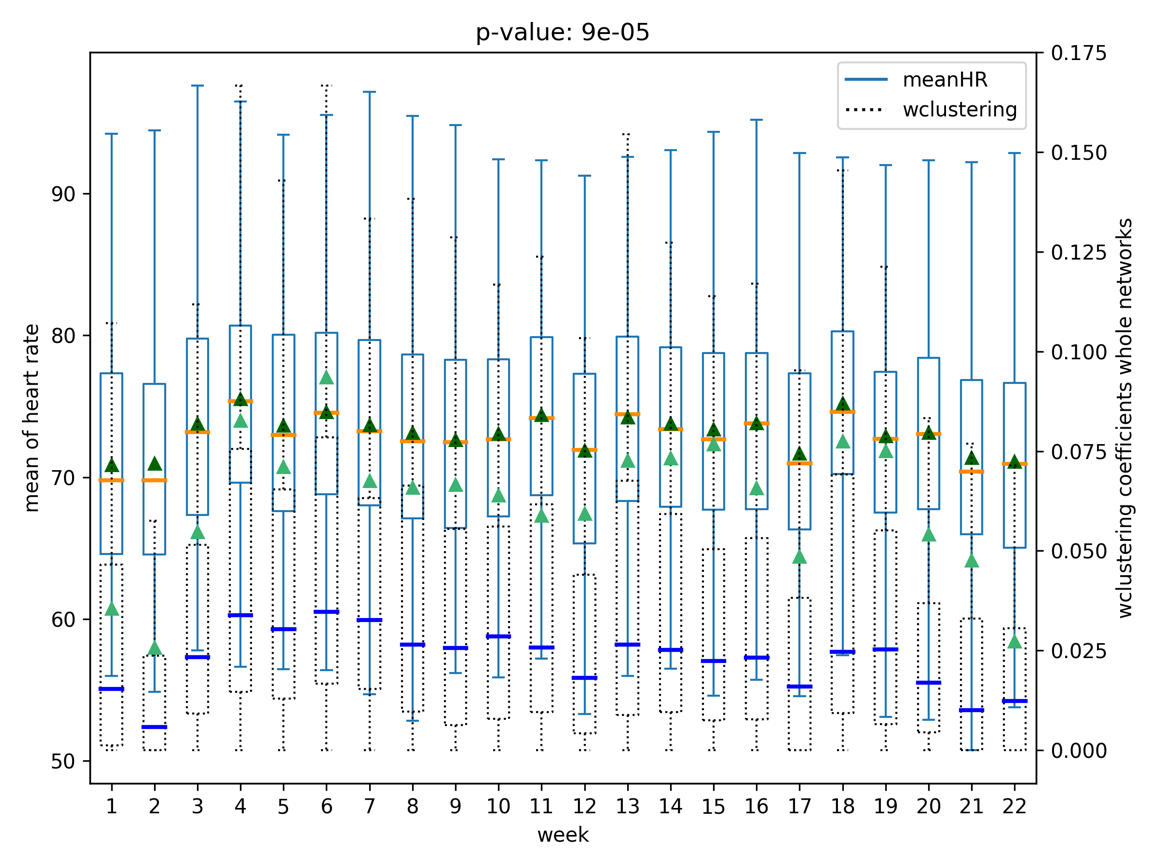


Figure 6 box plot of mean of heart rate and clustering coefficients of whole network


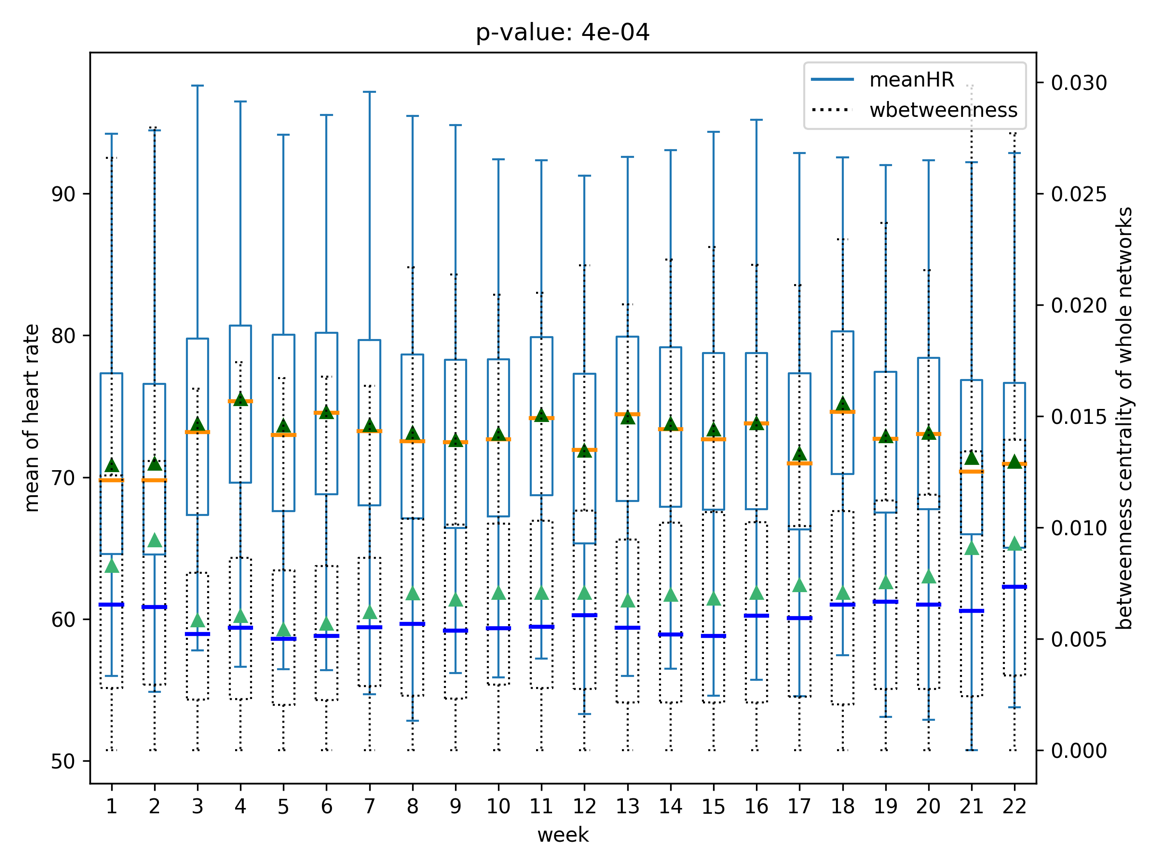


Figure 7 box plot of mean of heart rate and betweenness centrality of whole network


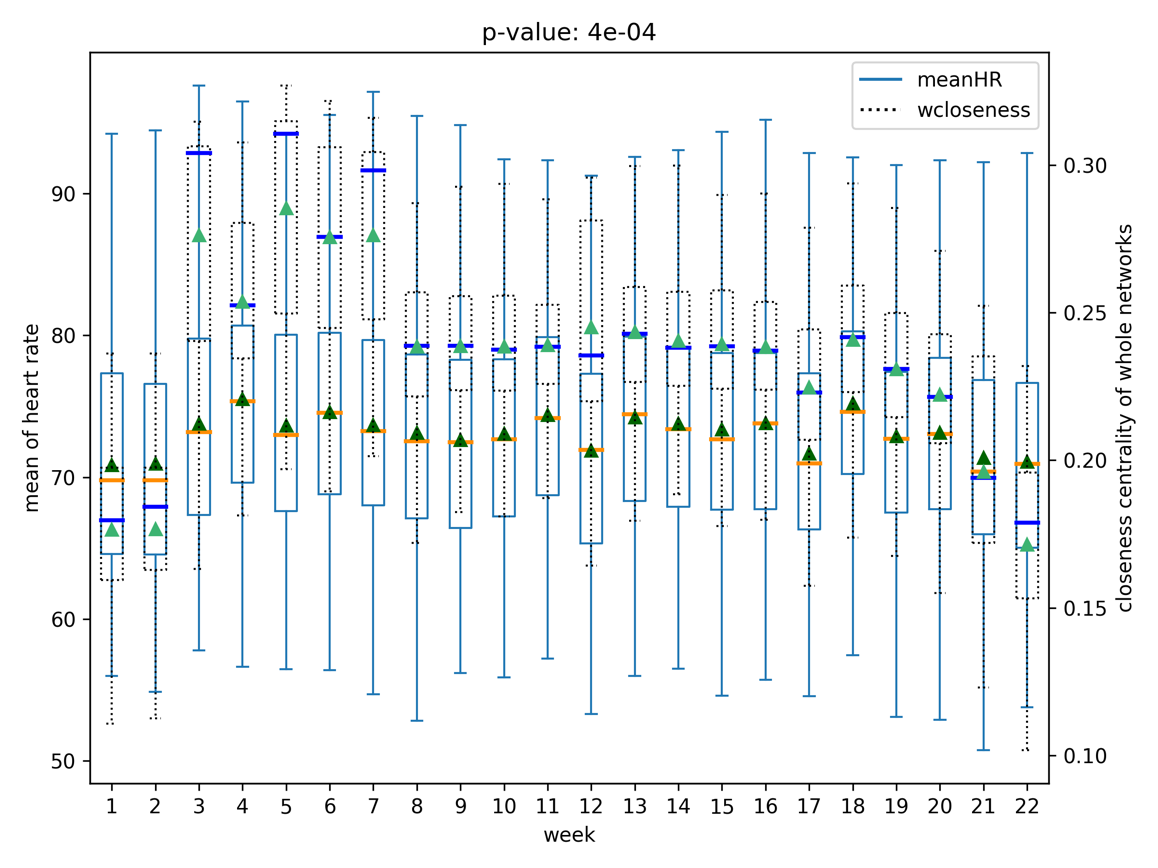


Figure 8 box plot of mean of heart rate and closeness centrality of whole network


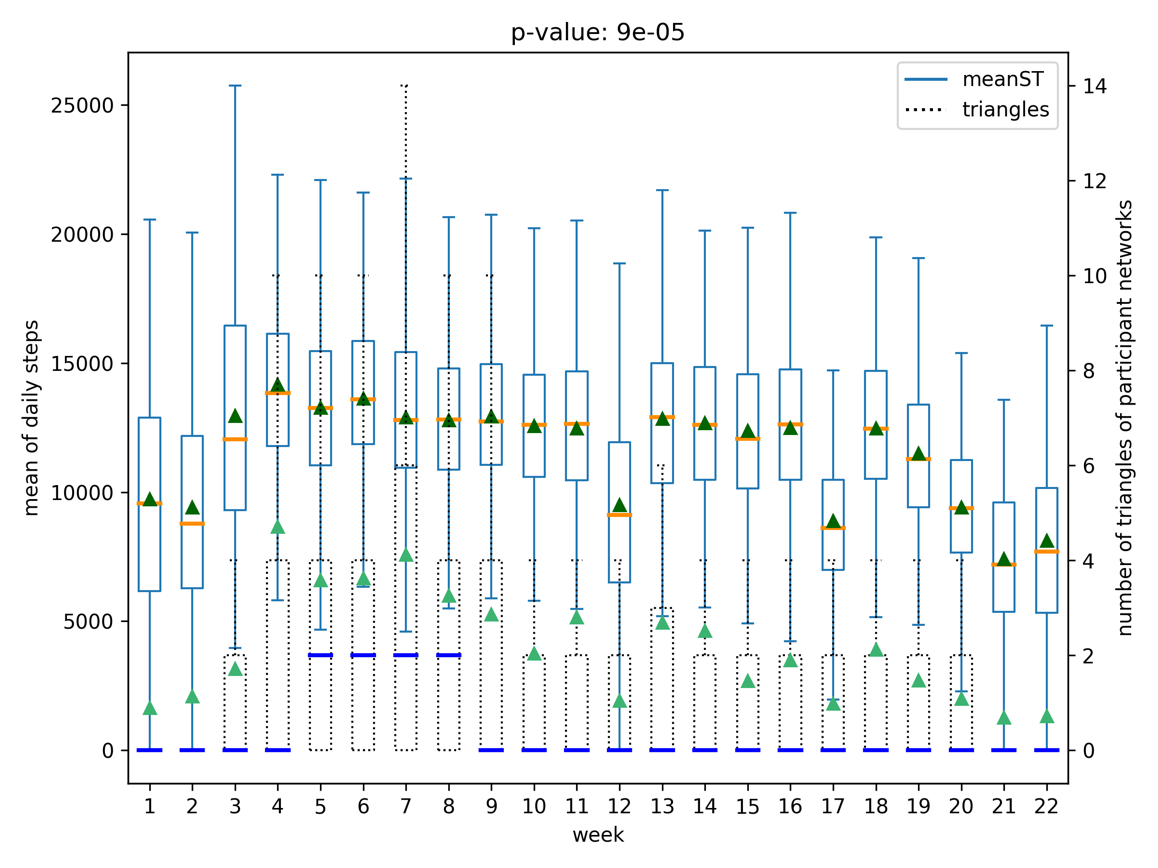


Figure 9 box plot of mean of daily steps and number of triangles of participant network


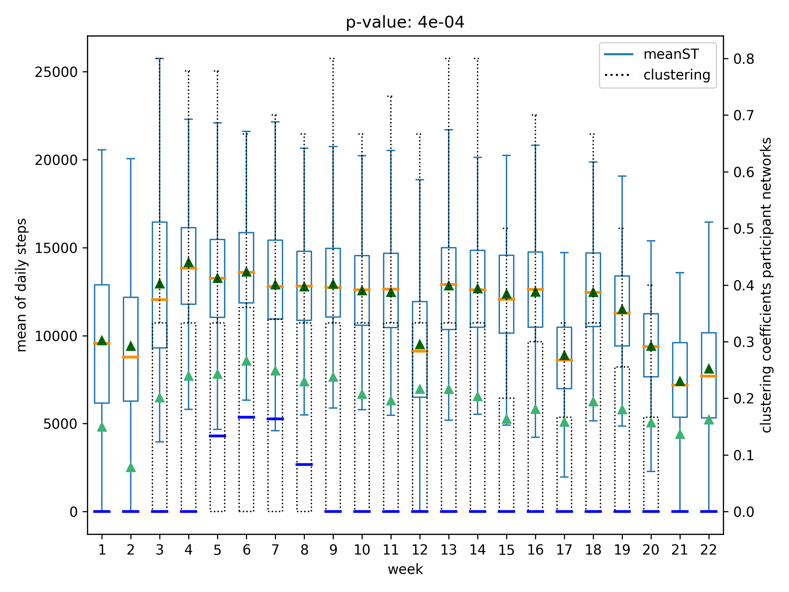


Figure 10 box plot of mean of daily steps and clustering coefficients of participant network


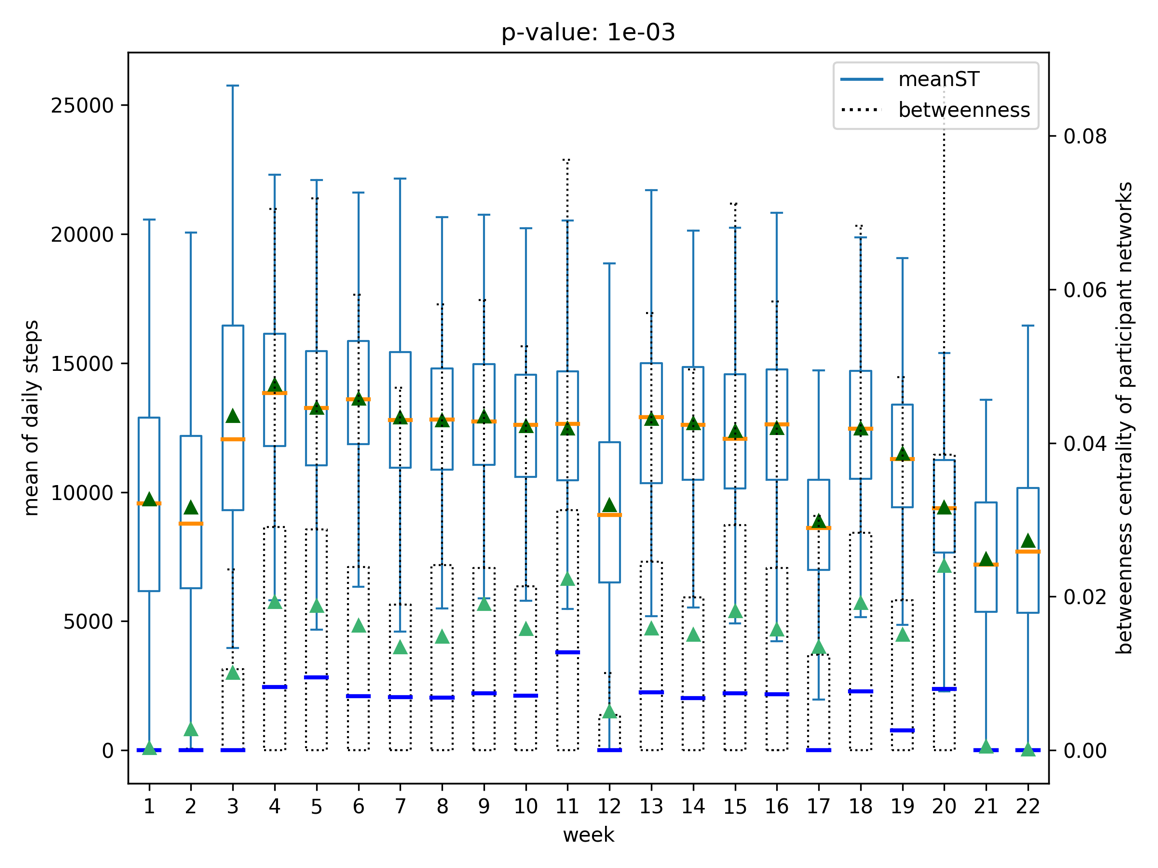


Figure 11 box plot of mean of daily steps and betweenness centrality of participant network


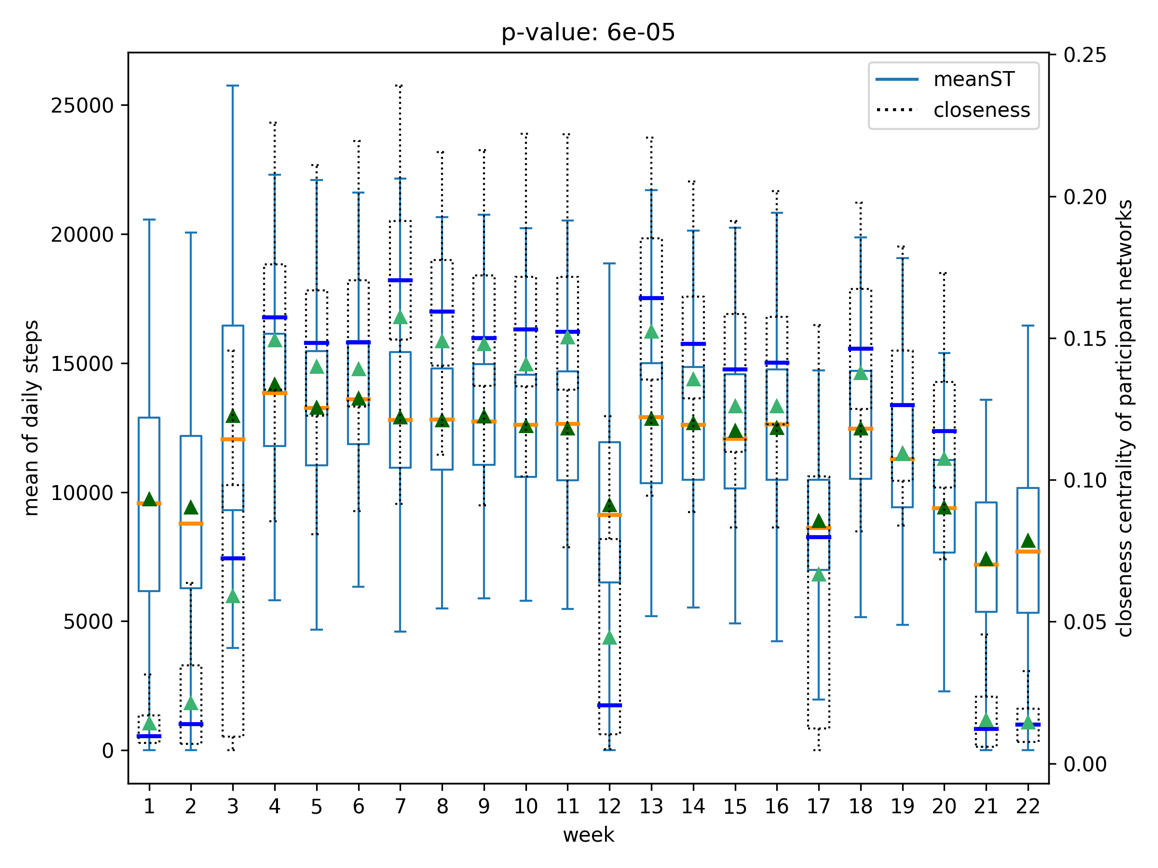


Figure 12 box plot of mean of daily steps and closeness centrality of participant network


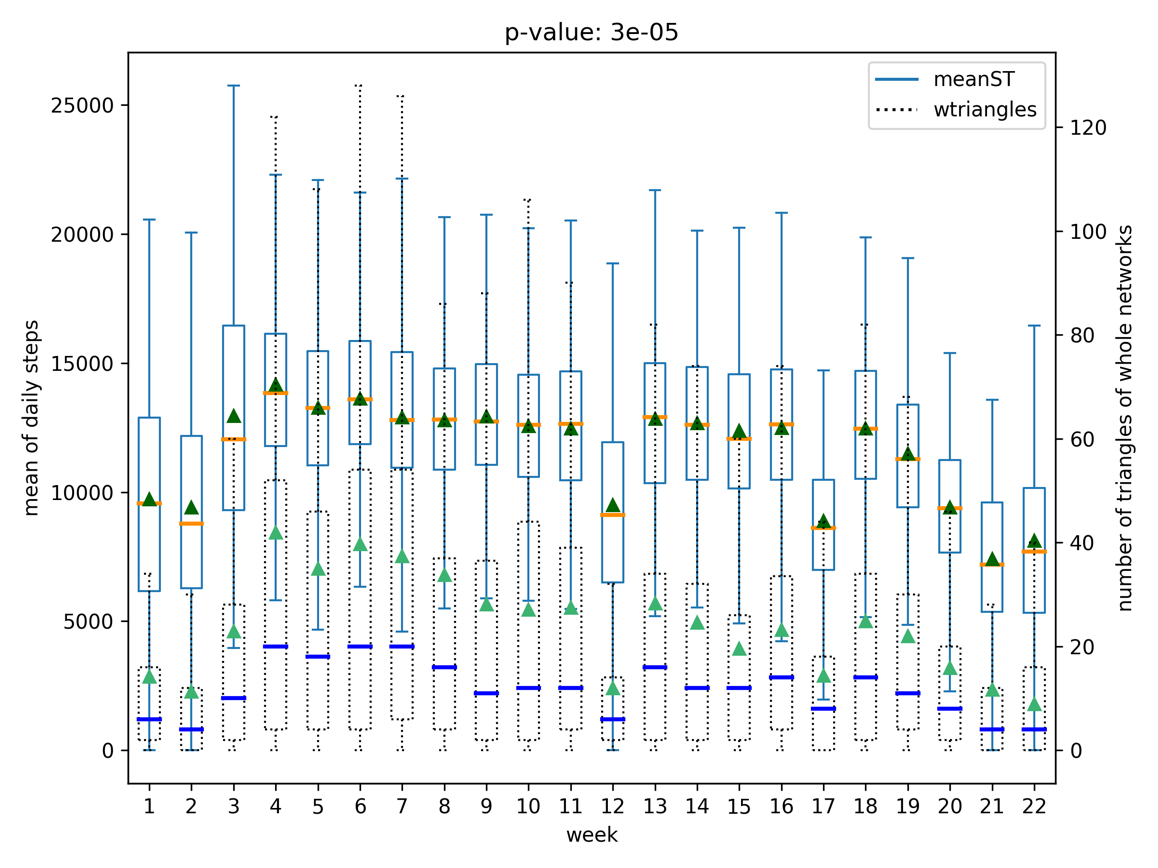


Figure 13 box plot of mean of daily steps and number of triangles of whole network


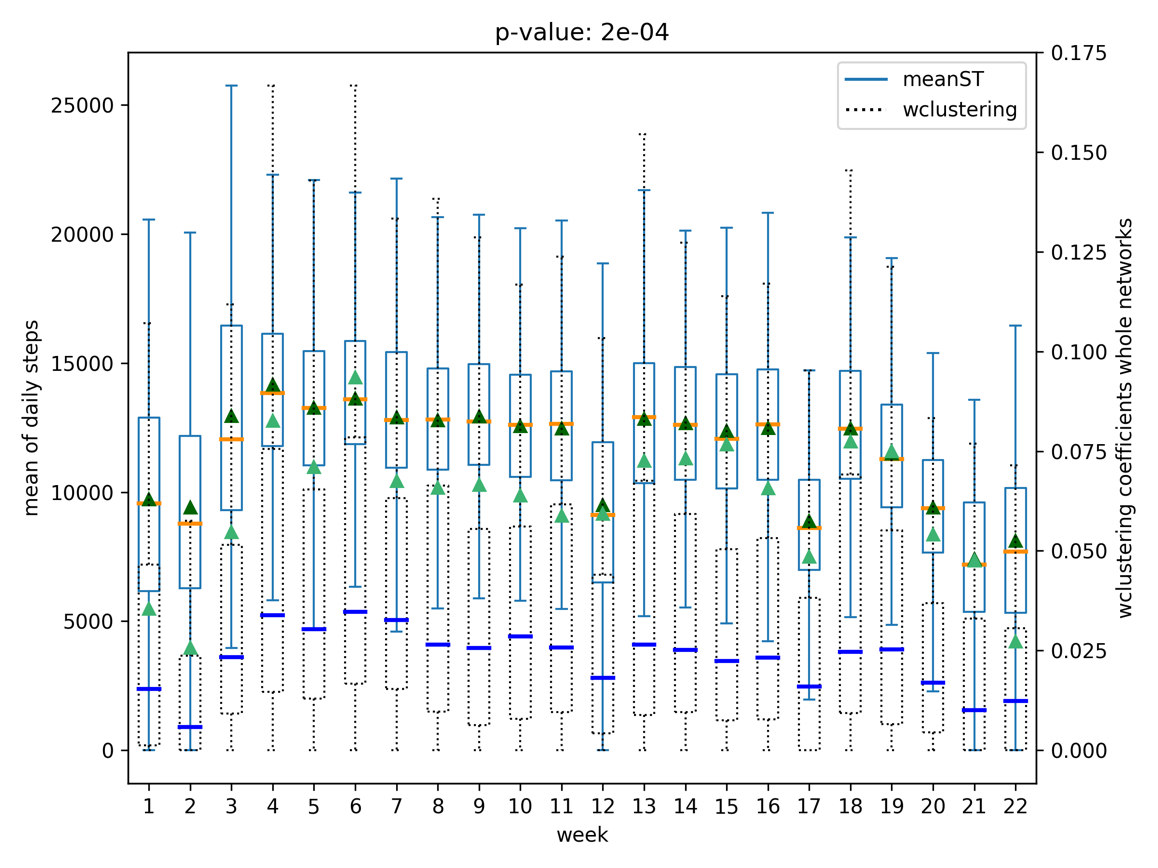


Figure 14 box plot of mean of daily steps and clustering coefficients of whole network


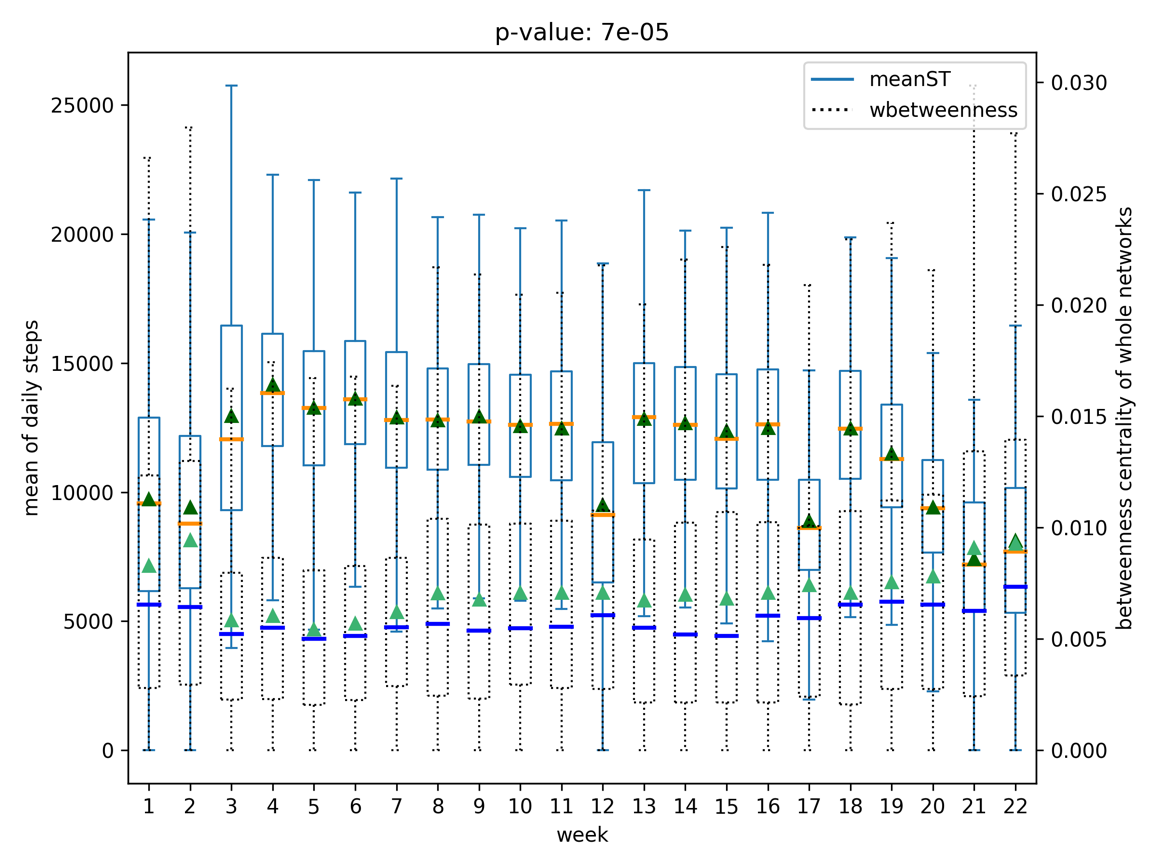


Figure 15 box plot of mean of daily steps and betweenness centrality of whole network


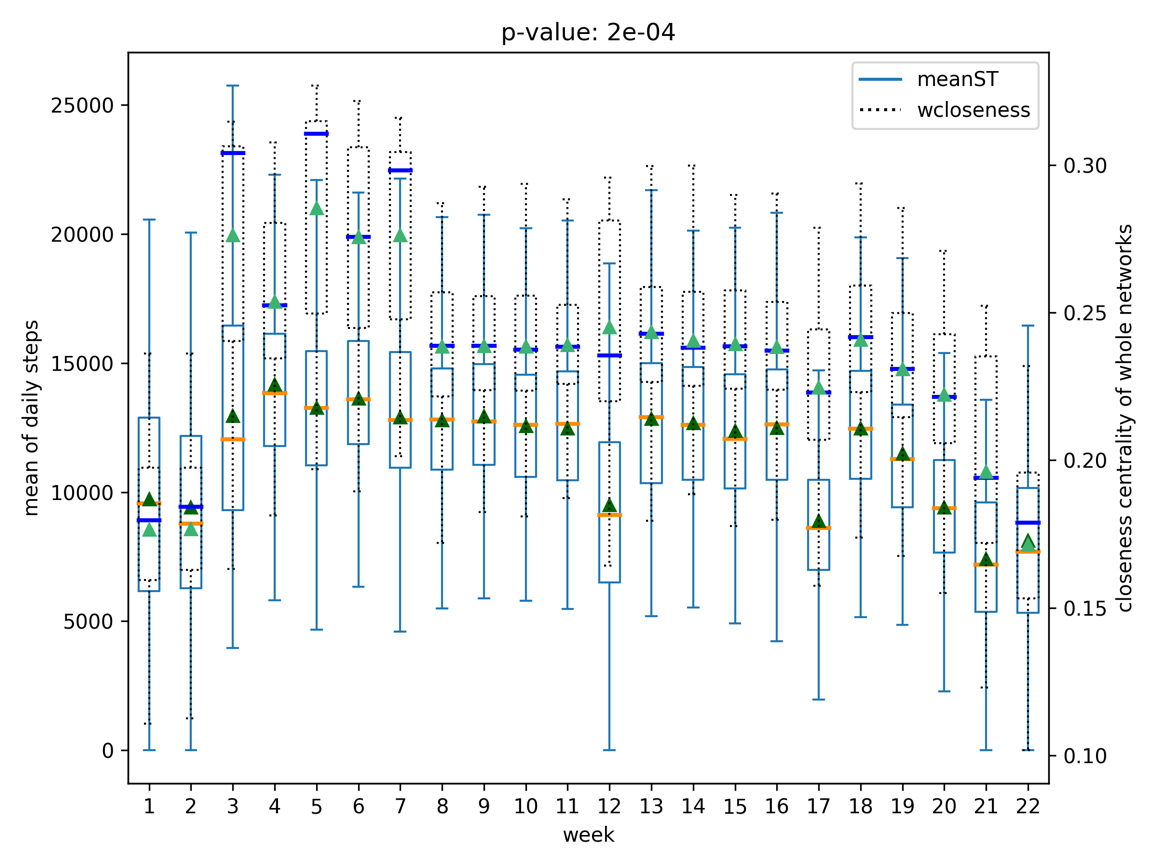


Figure 16 box plot of mean of daily steps and closeness centrality of whole network


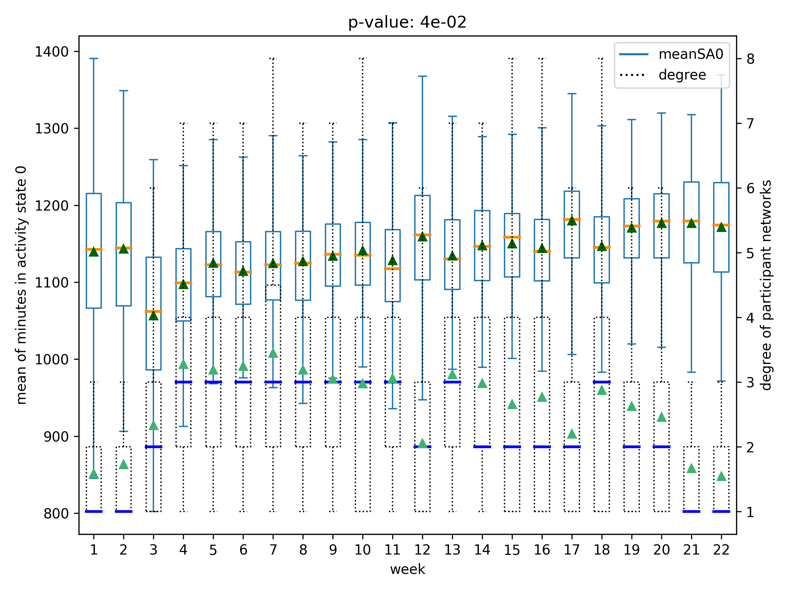


Figure 17 box plot of mean of minutes in state 0 and degree of participant network


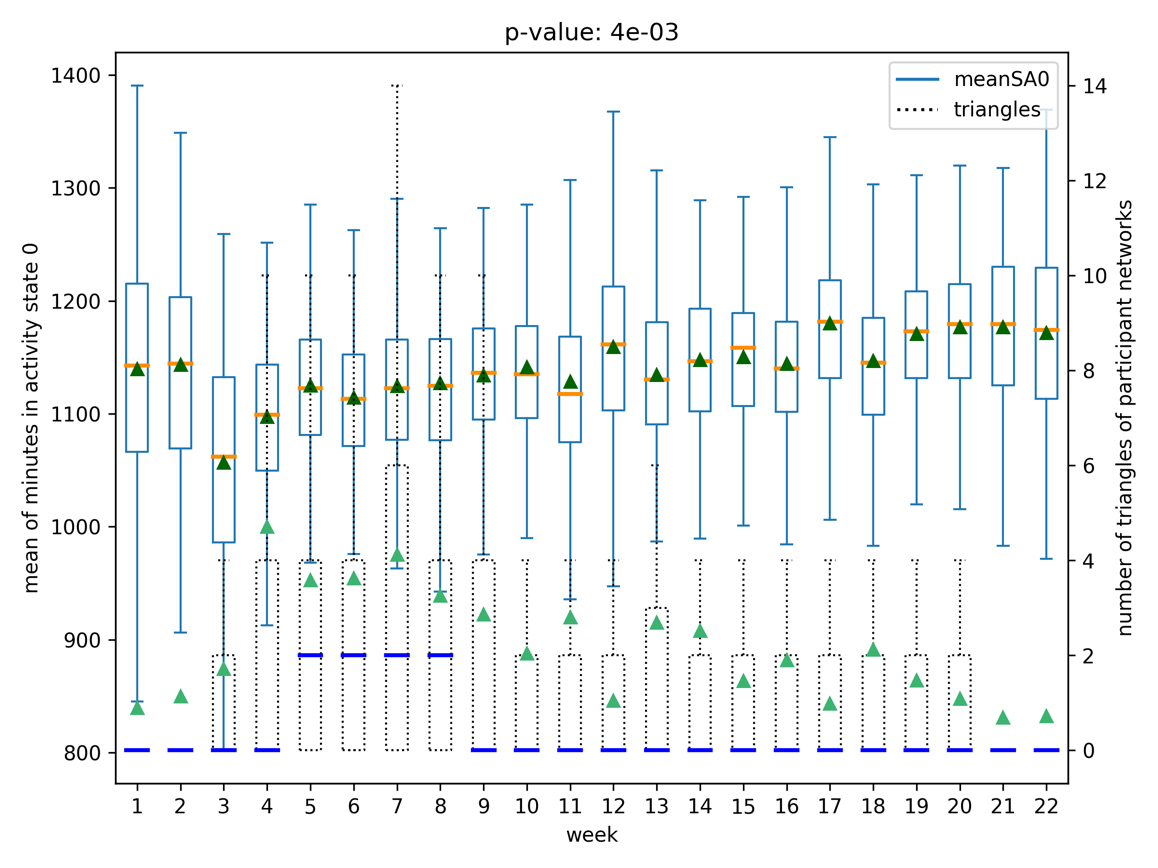


Figure 18 box plot of mean of minutes in state 0 and number of triangles of participant network


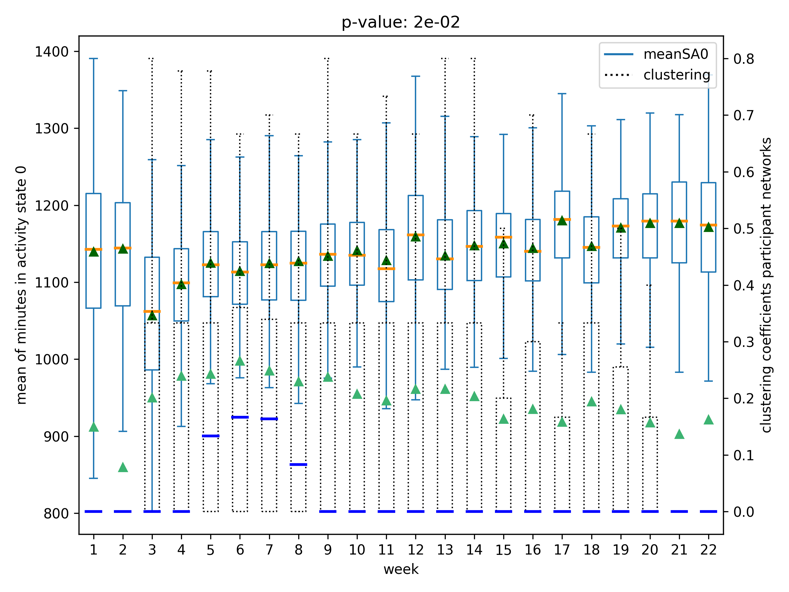


Figure 19 box plot of mean of minutes in state 0 and clustering coefficients of participant network


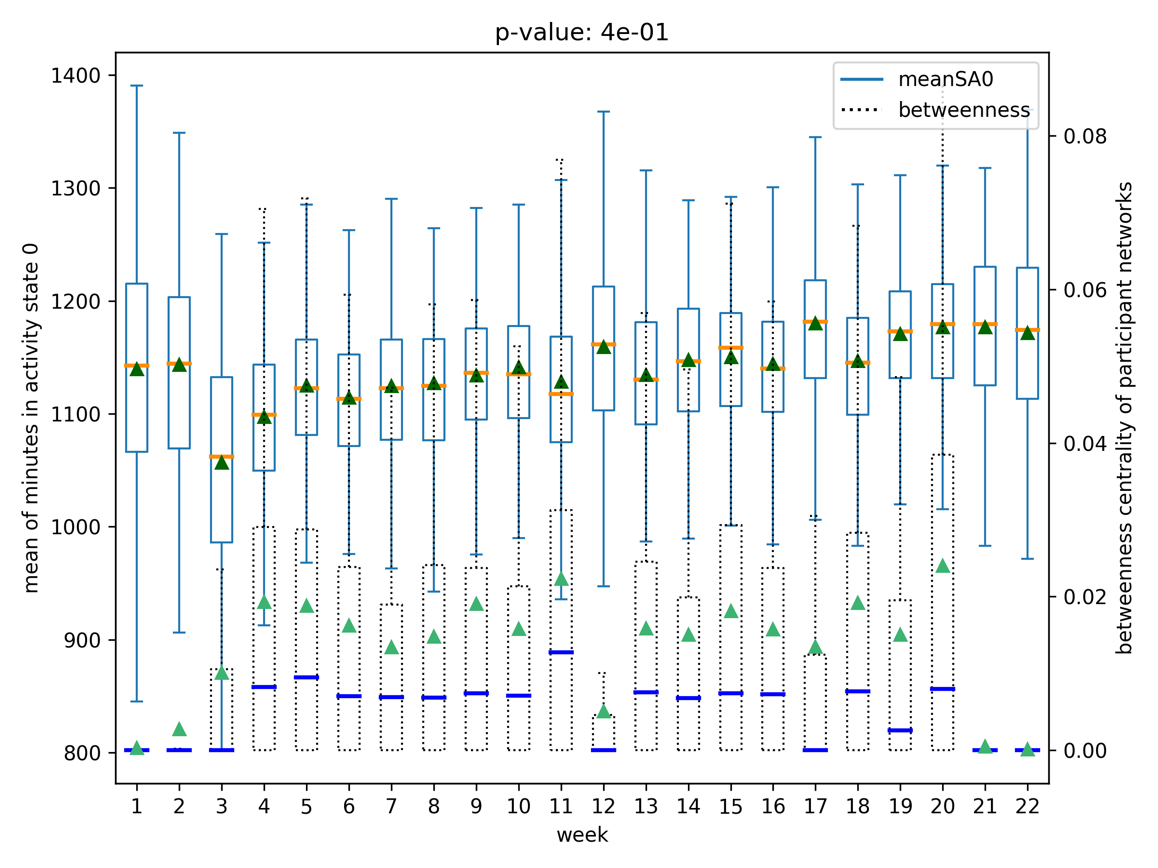


Figure 20 box plot of mean of minutes in state 0 and betweenness centrality of participant network


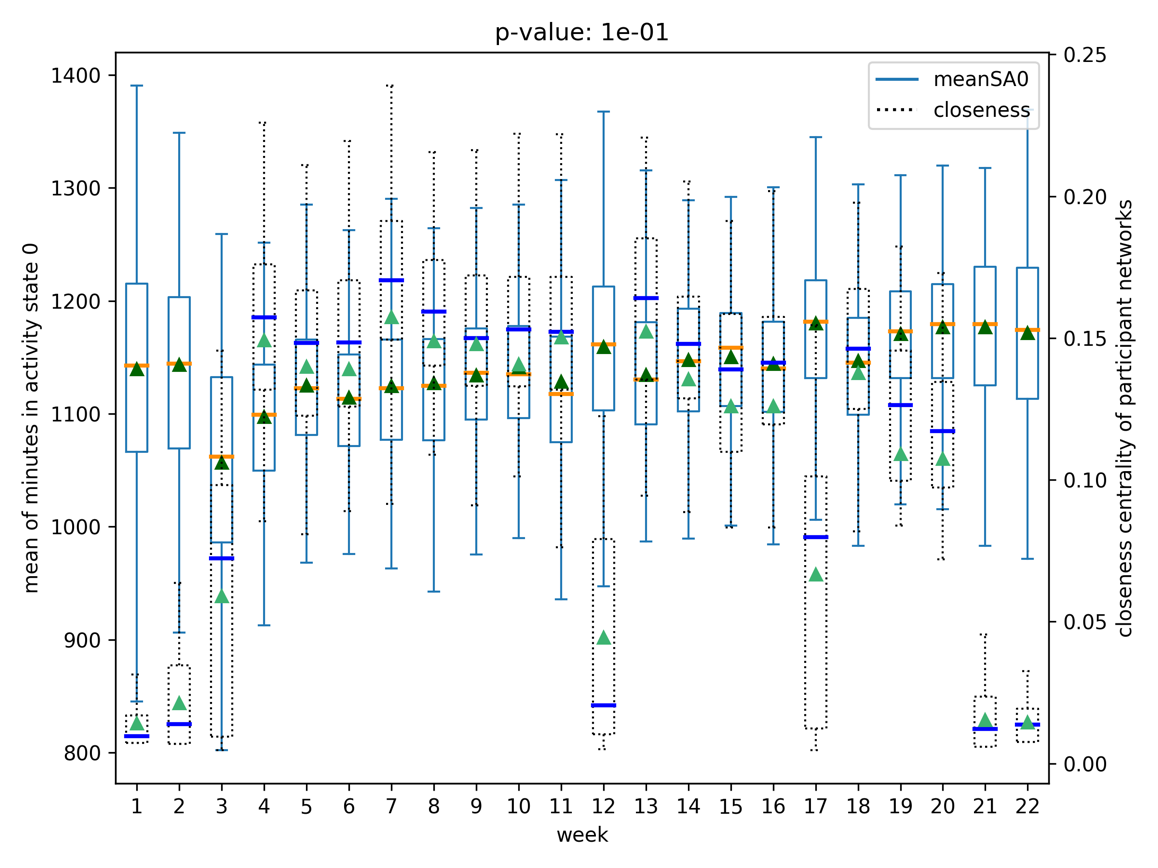


Figure 21 box plot of mean of minutes in state 0 and closeness centrality of participant network


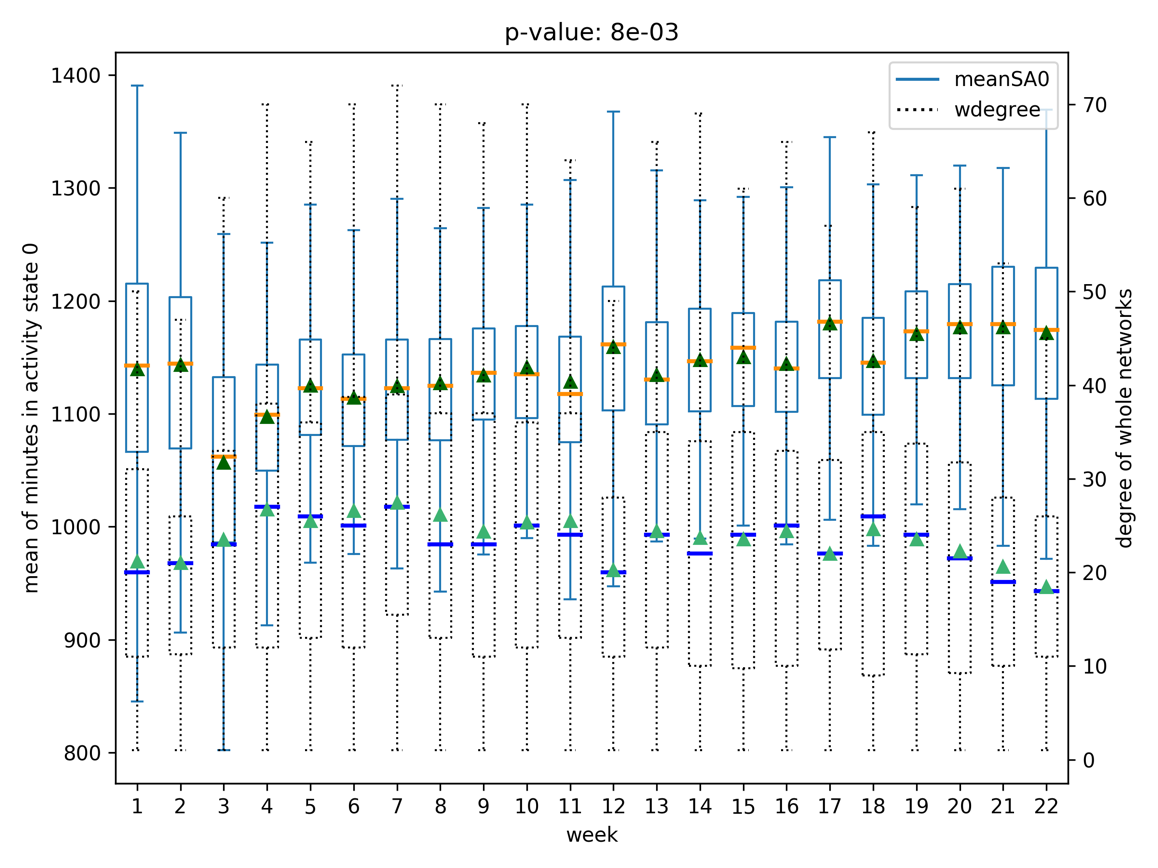


Figure 22 box plot of mean of minutes in state 0 and degree of whole network


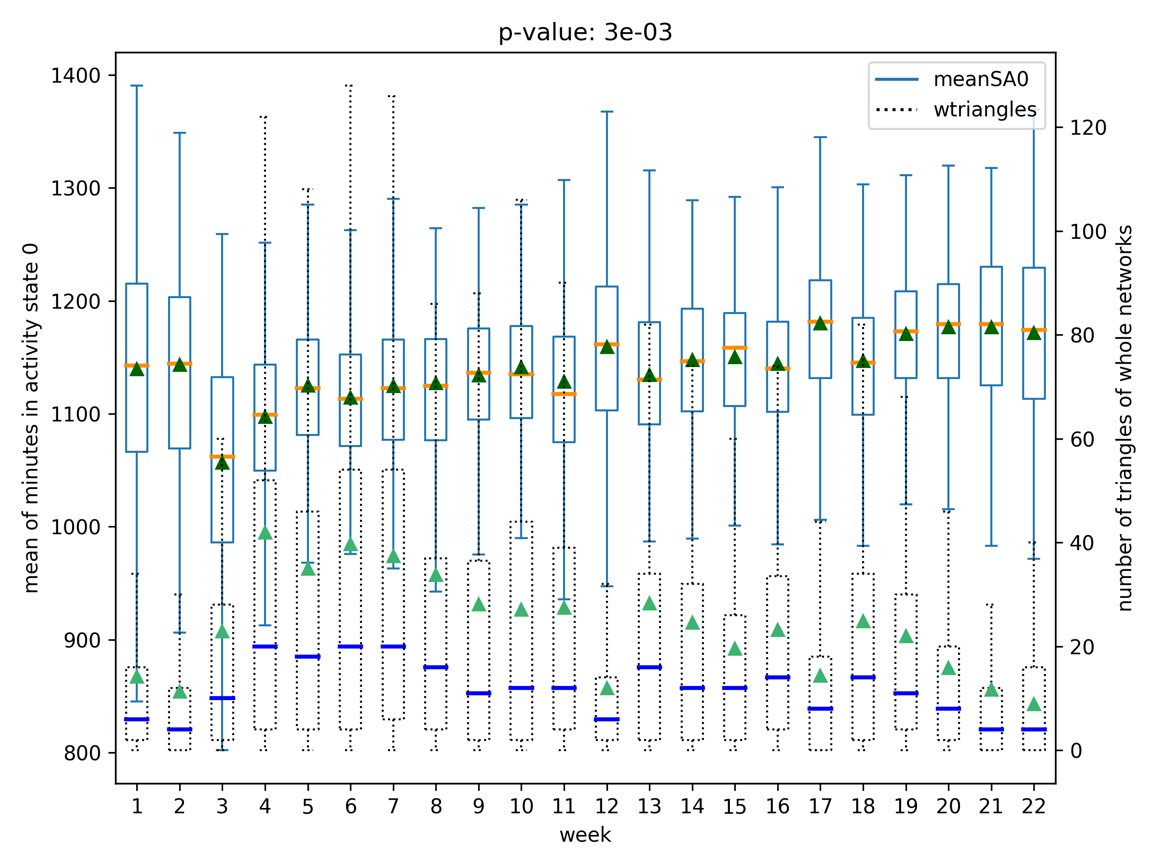


Figure 23 box plot of mean of minutes in state 0 and number of triangles of whole network


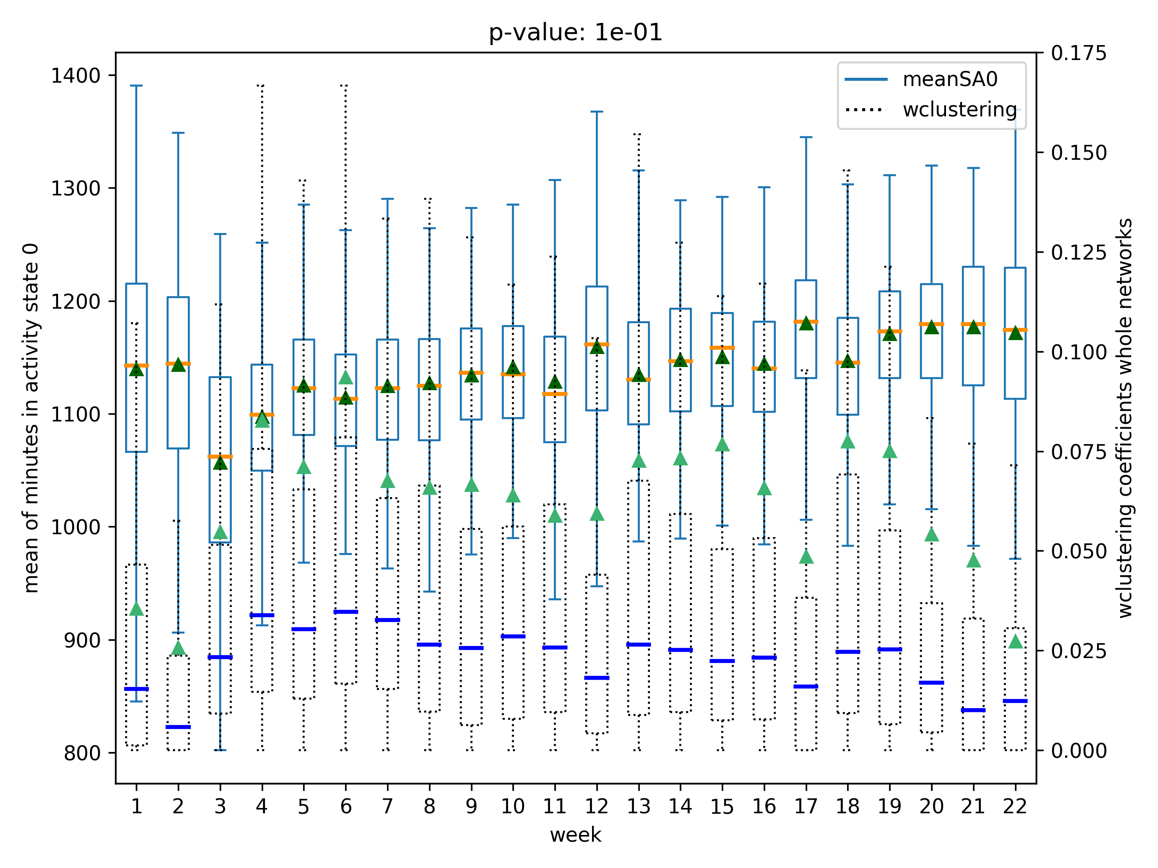


Figure 24 box plot of mean of minutes in state 0 and clustering coefficients of whole network


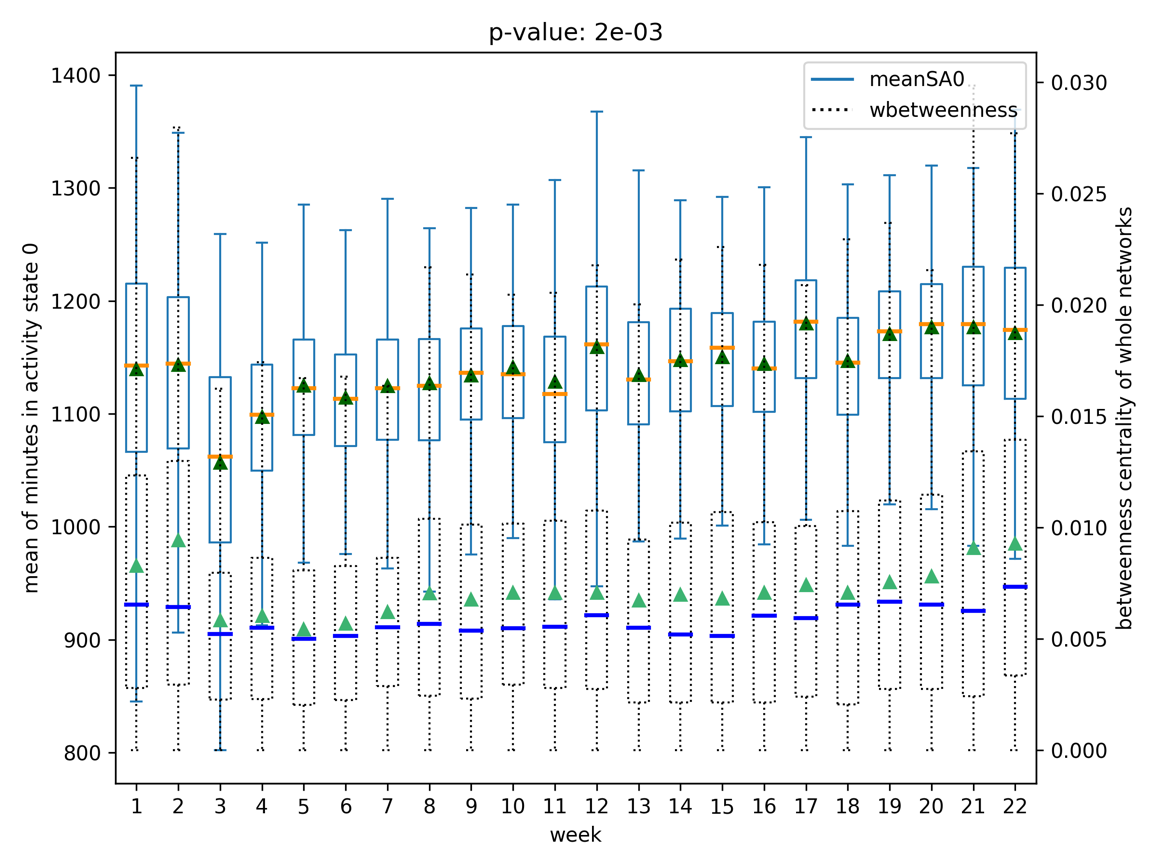


Figure 25 box plot of mean of minutes in state 0 and betweenness centrality of whole network


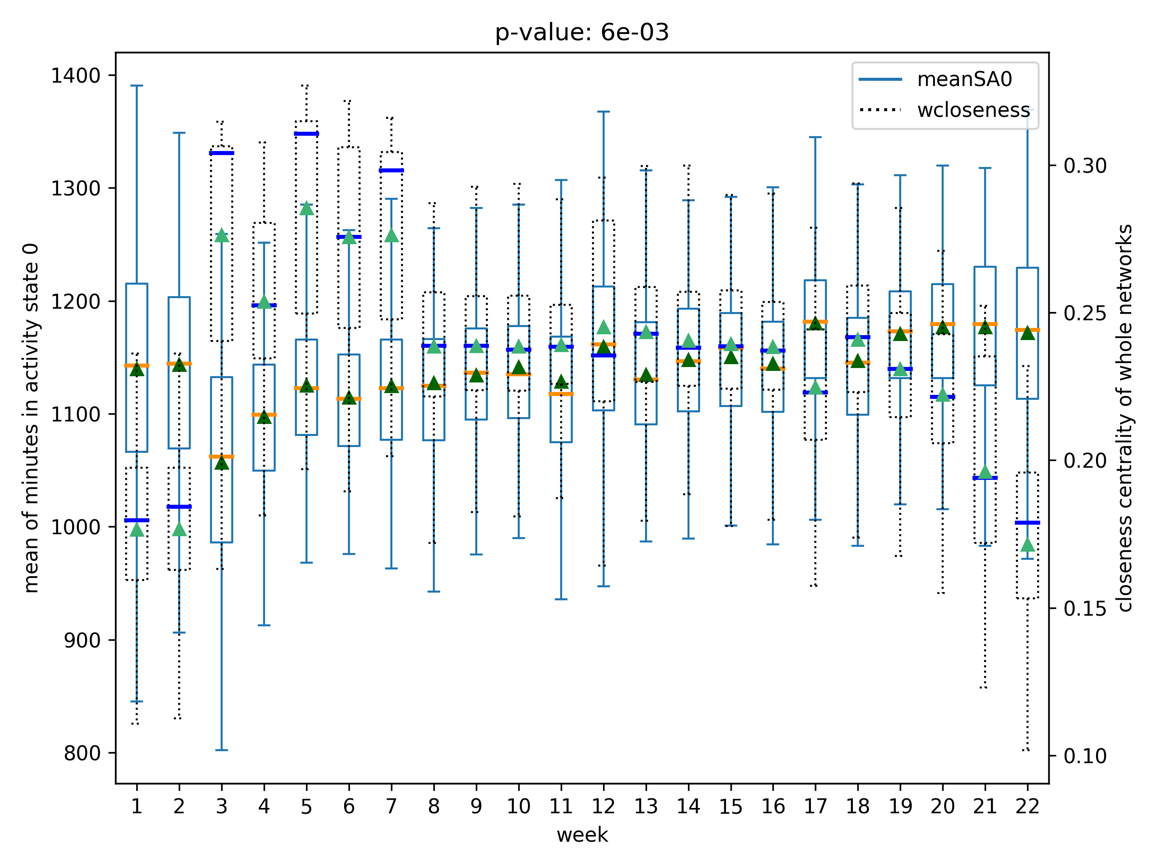


Figure 26 box plot of mean of minutes in state 0 and closeness centrality of whole network


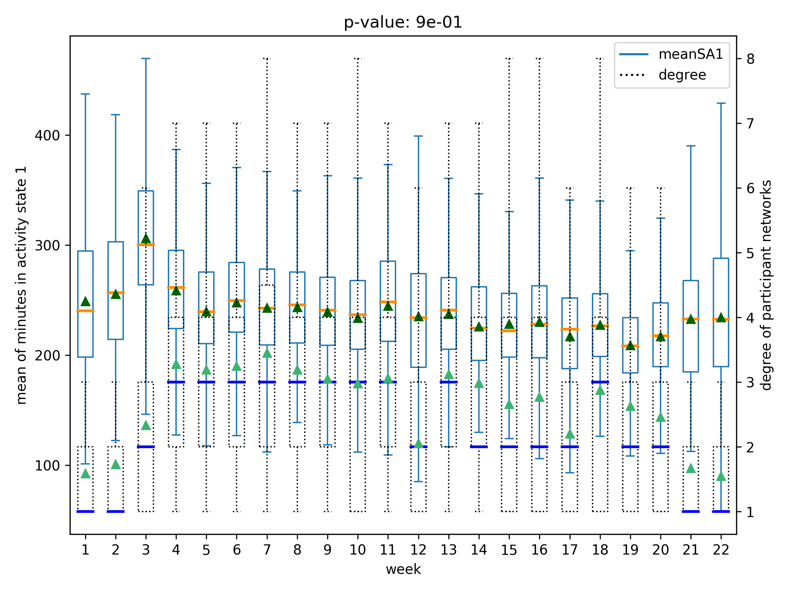


Figure 27 box plot of mean of minutes in state 1 and degree of participant network


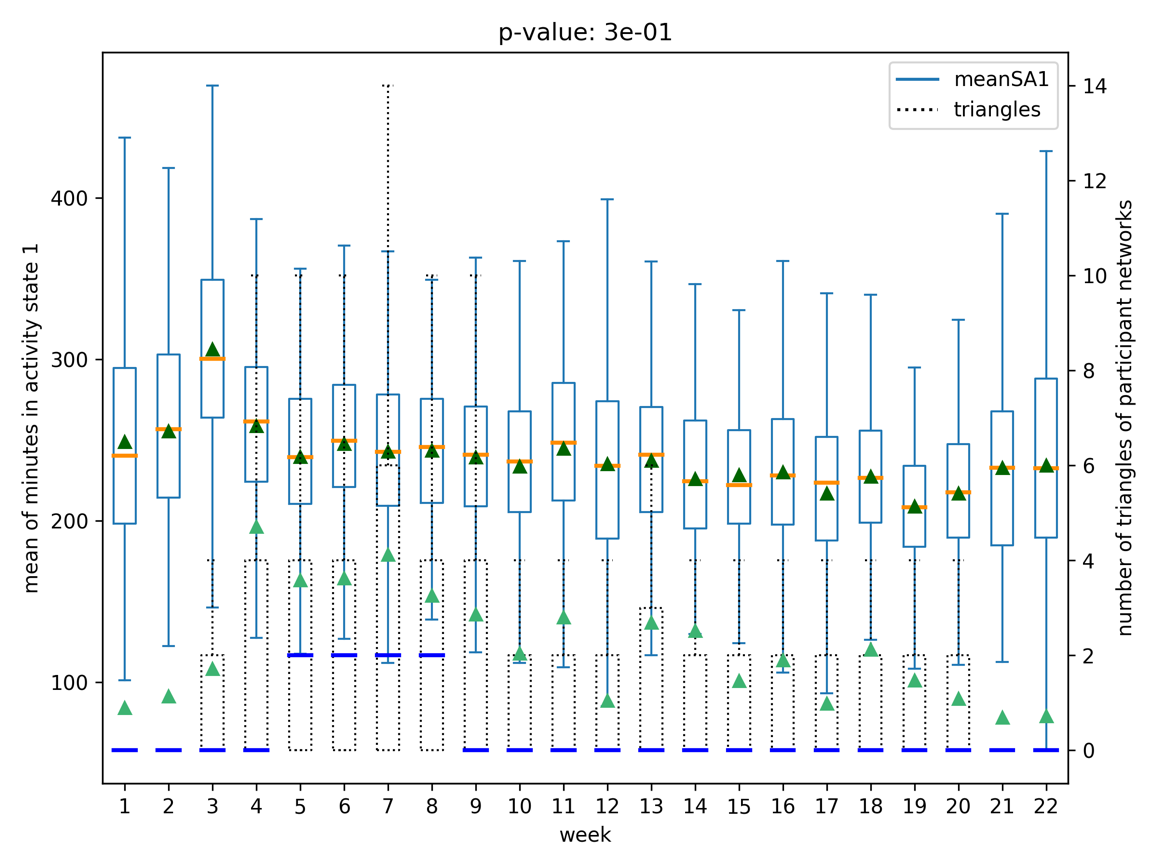


Figure 28 box plot of mean of minutes in state 1 and number of triangles of participant network


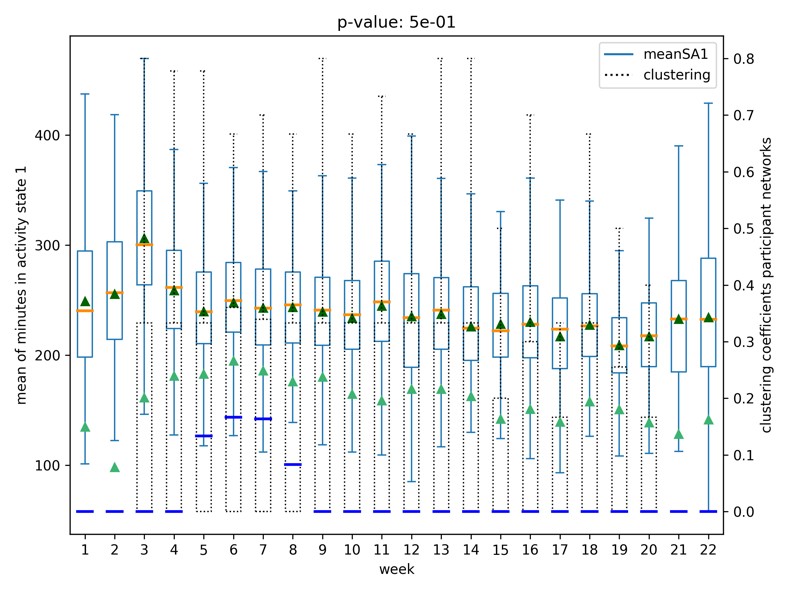


Figure 29 box plot of mean of minutes in state 1 and clustering coefficients of participant network


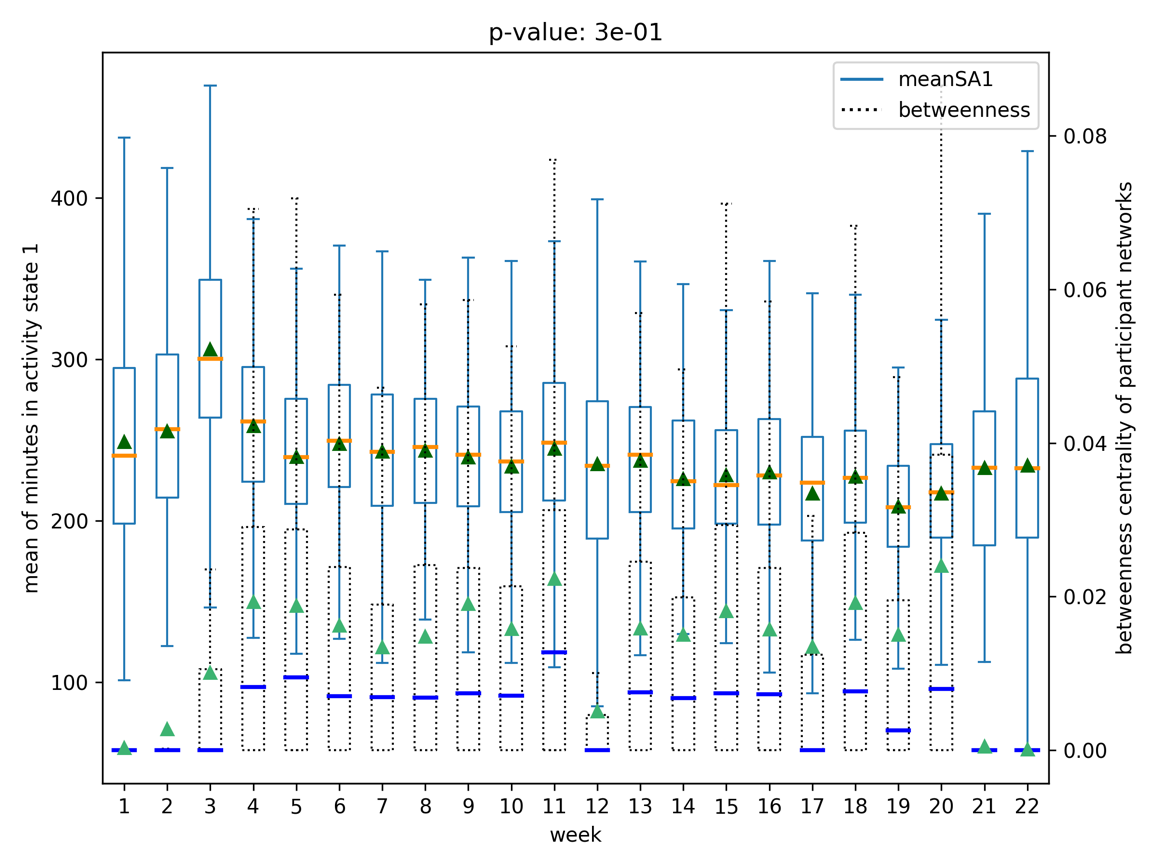


Figure 30 box plot of mean of minutes in state 1 and betweenness centrality of participant network


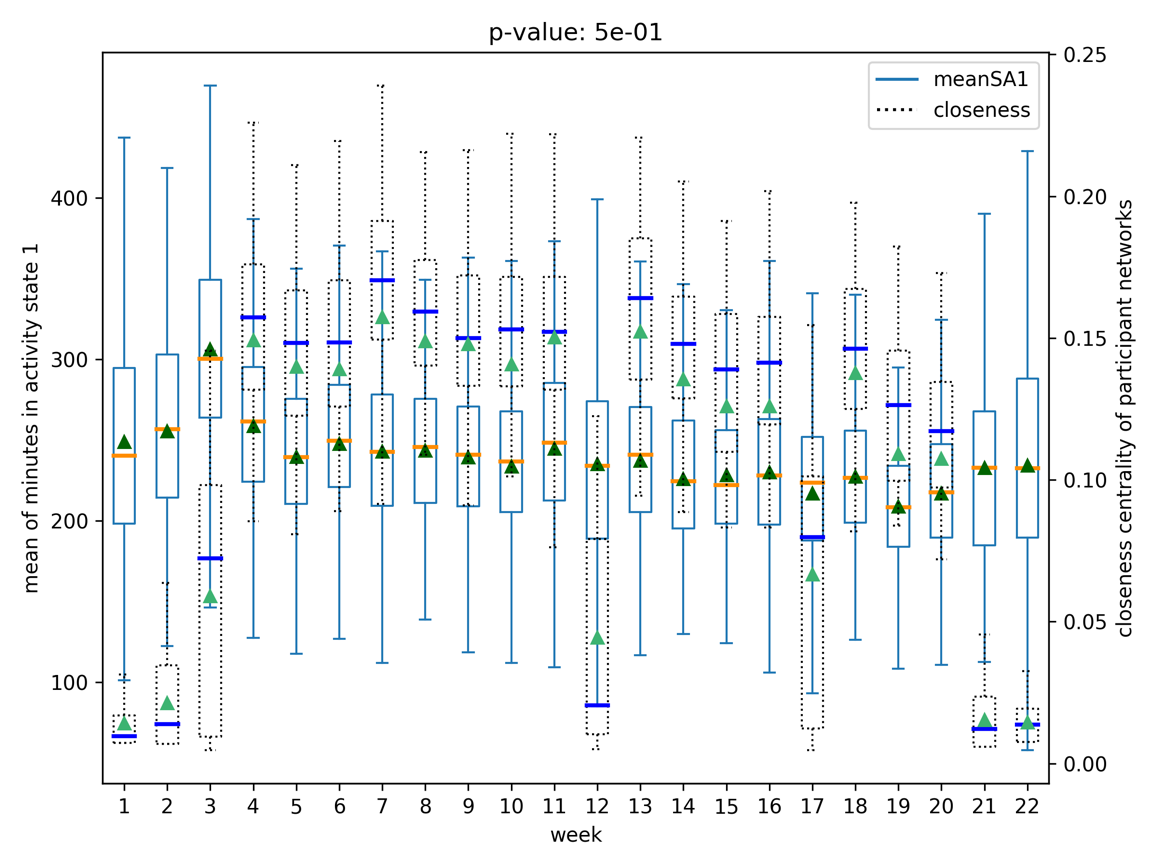


Figure 31 box plot of mean of minutes in state 1 and closeness centrality of participant network


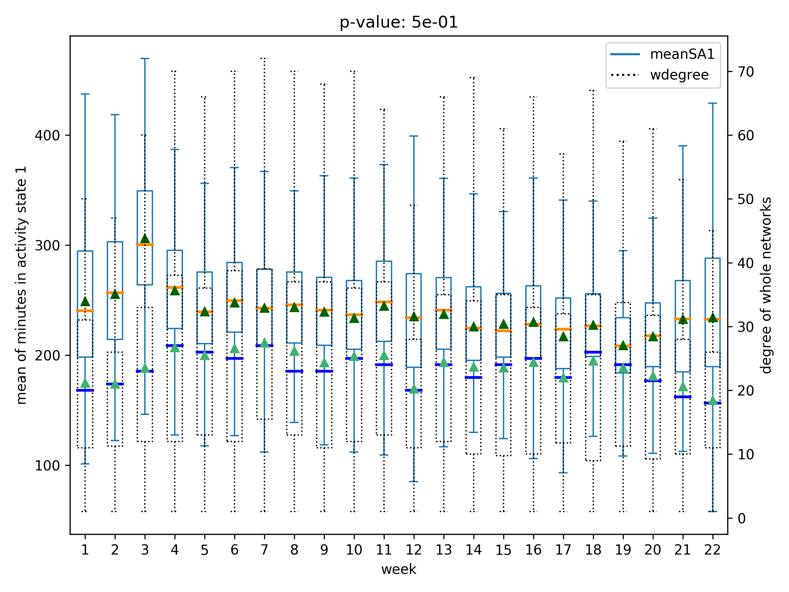


Figure 32 box plot of mean of minutes in state 1 and degree of whole network


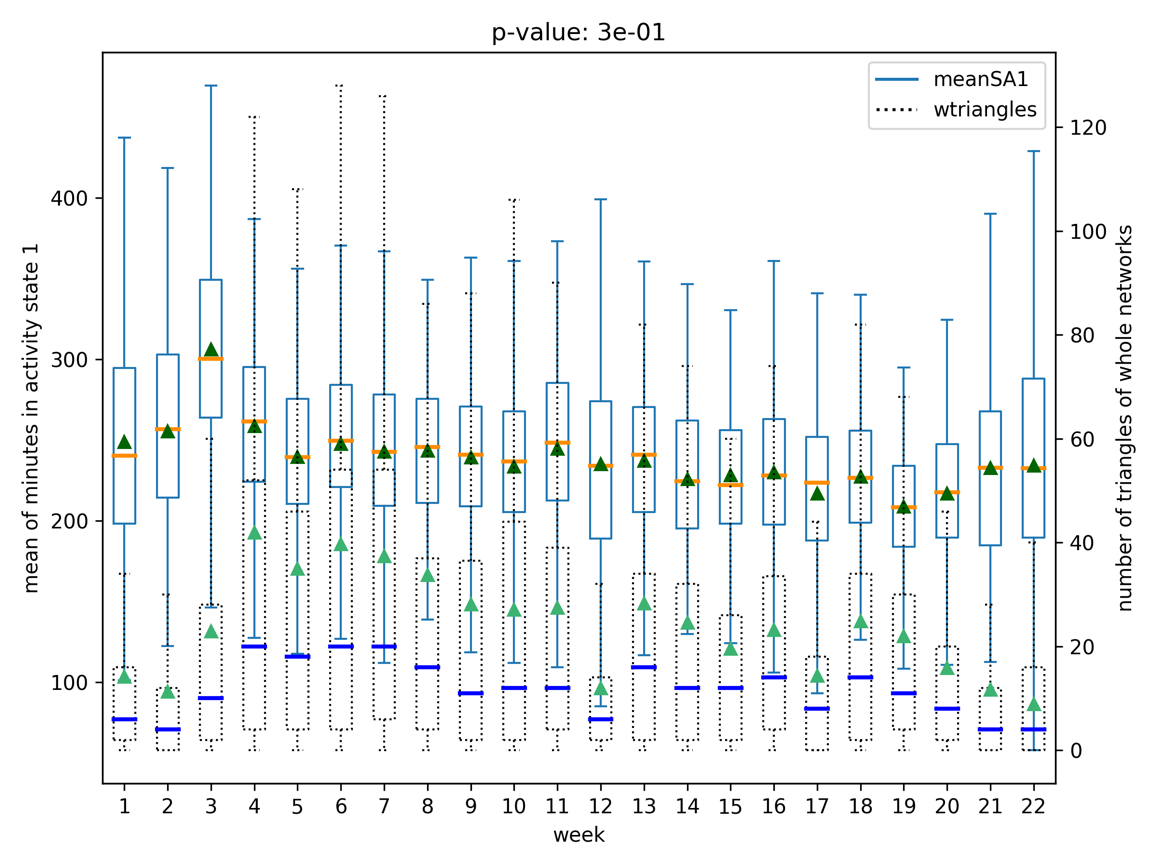


Figure 33 box plot of mean of minutes in state 1 and number of triangles of whole network


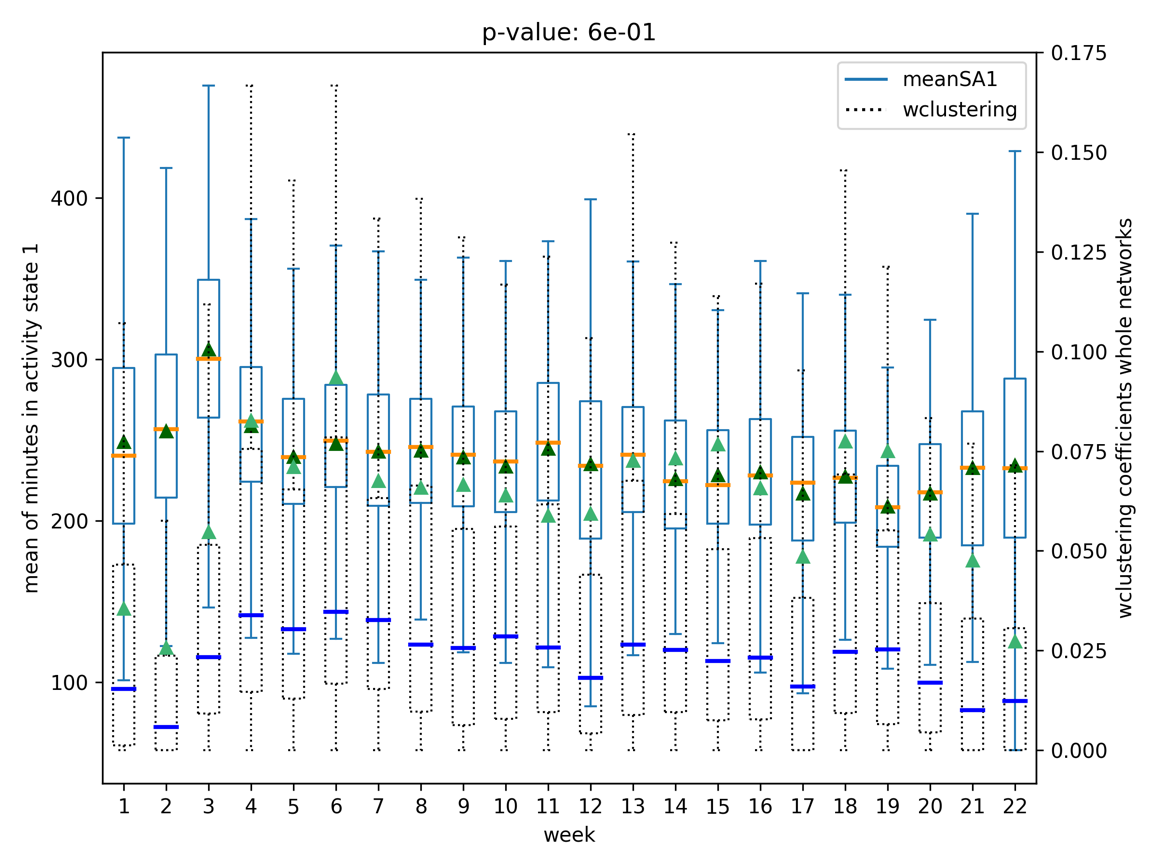


Figure 34 box plot of mean of minutes in state 1 and clustering coefficients of whole network


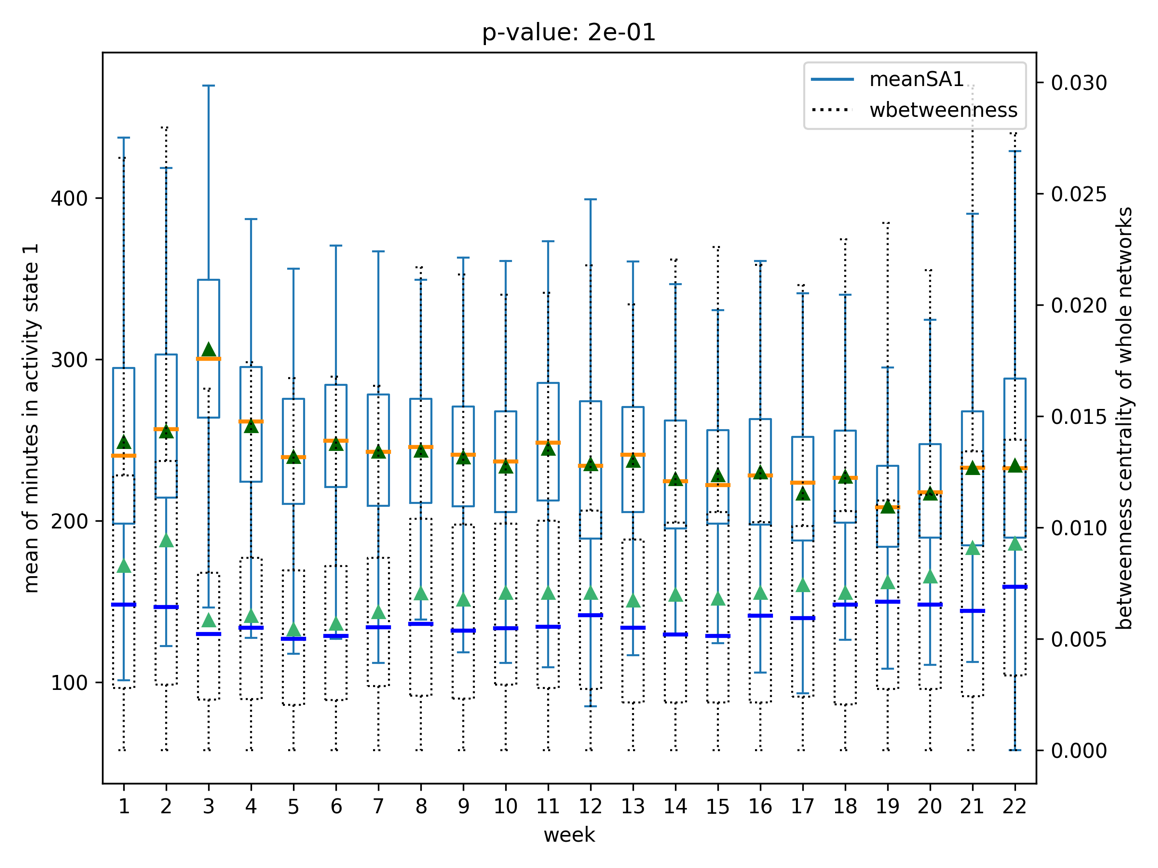


Figure 35 box plot of mean of minutes in state 1 and betweenness centrality of whole network


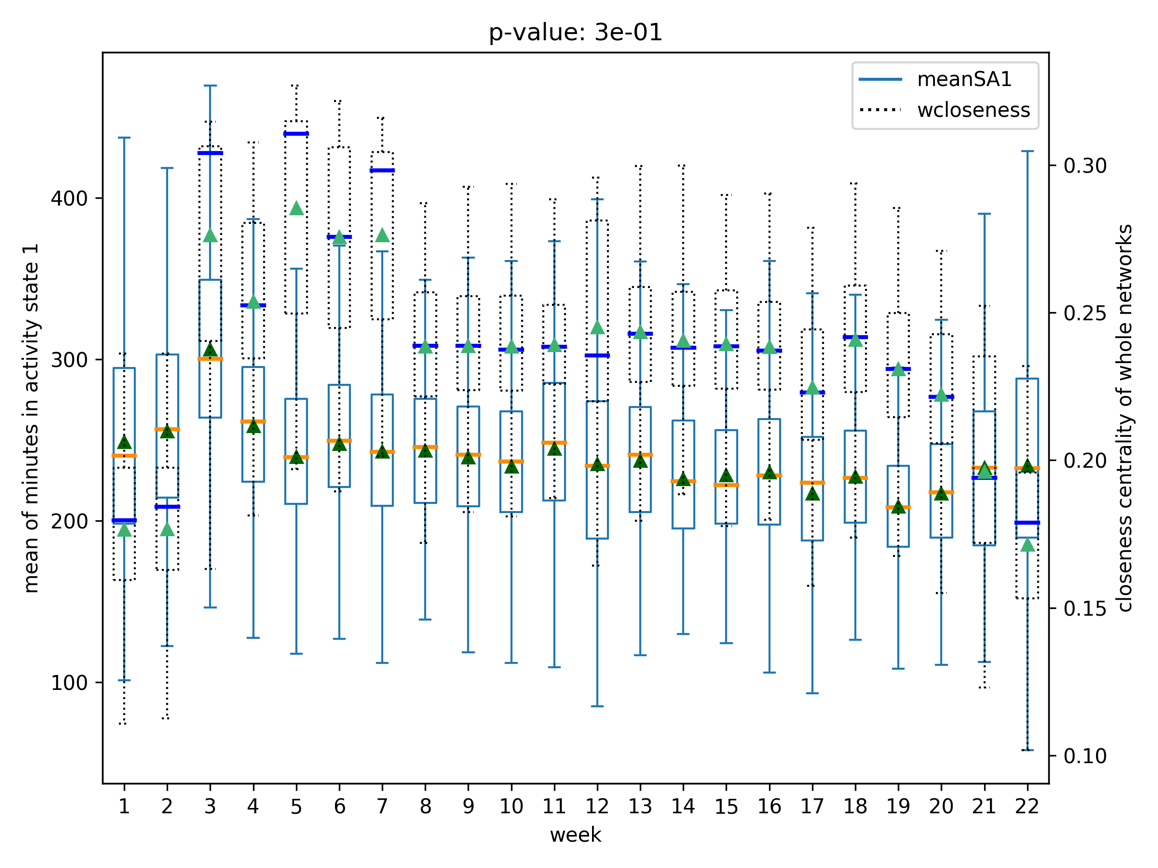


Figure 36 box plot of mean of minutes in state 1 and closeness centrality of whole network


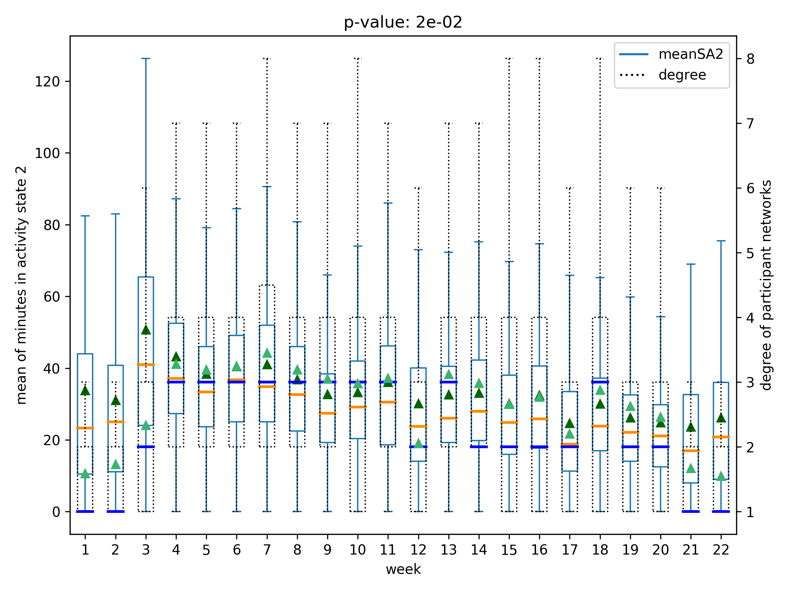


Figure 37 box plot of mean of minutes in state 2 and degree of participant network


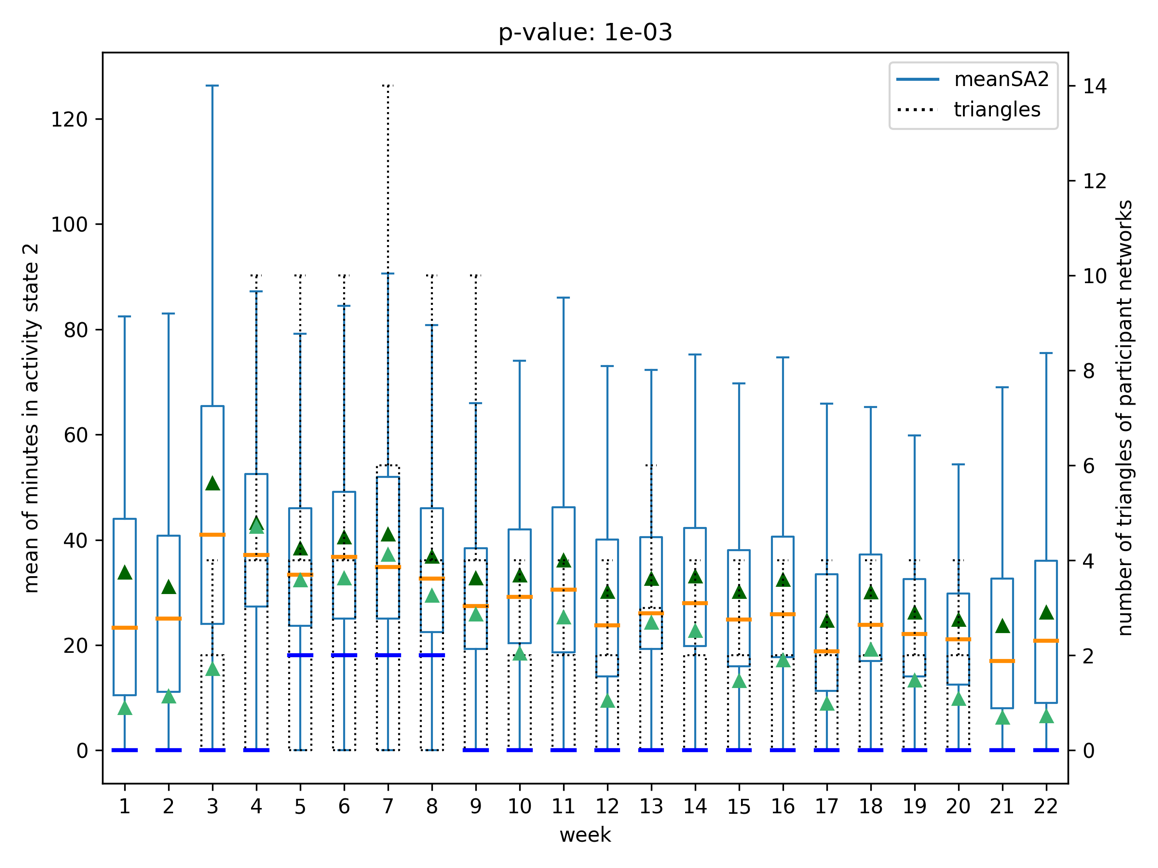


Figure 38 box plot of mean of minutes in state 2 and number of triangles of participant network


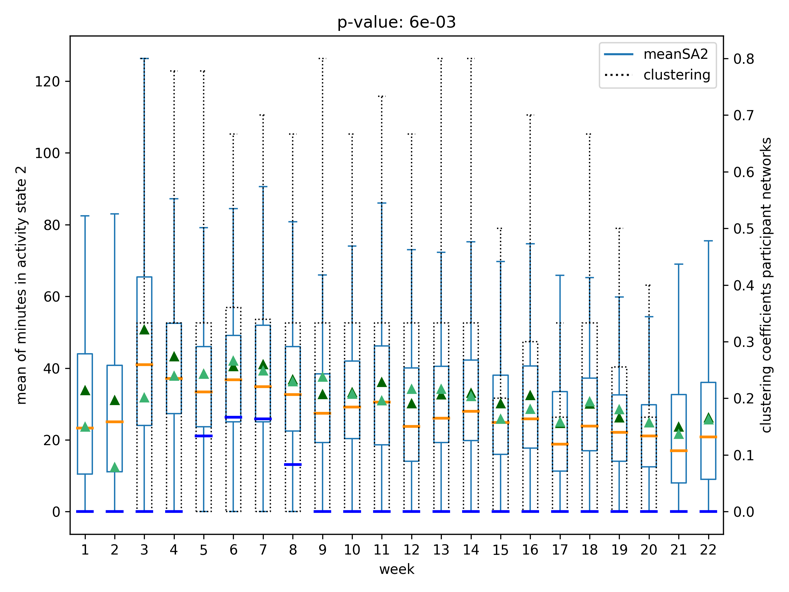


Figure 39 box plot of mean of minutes in state 2 and clustering coefficients of participant network


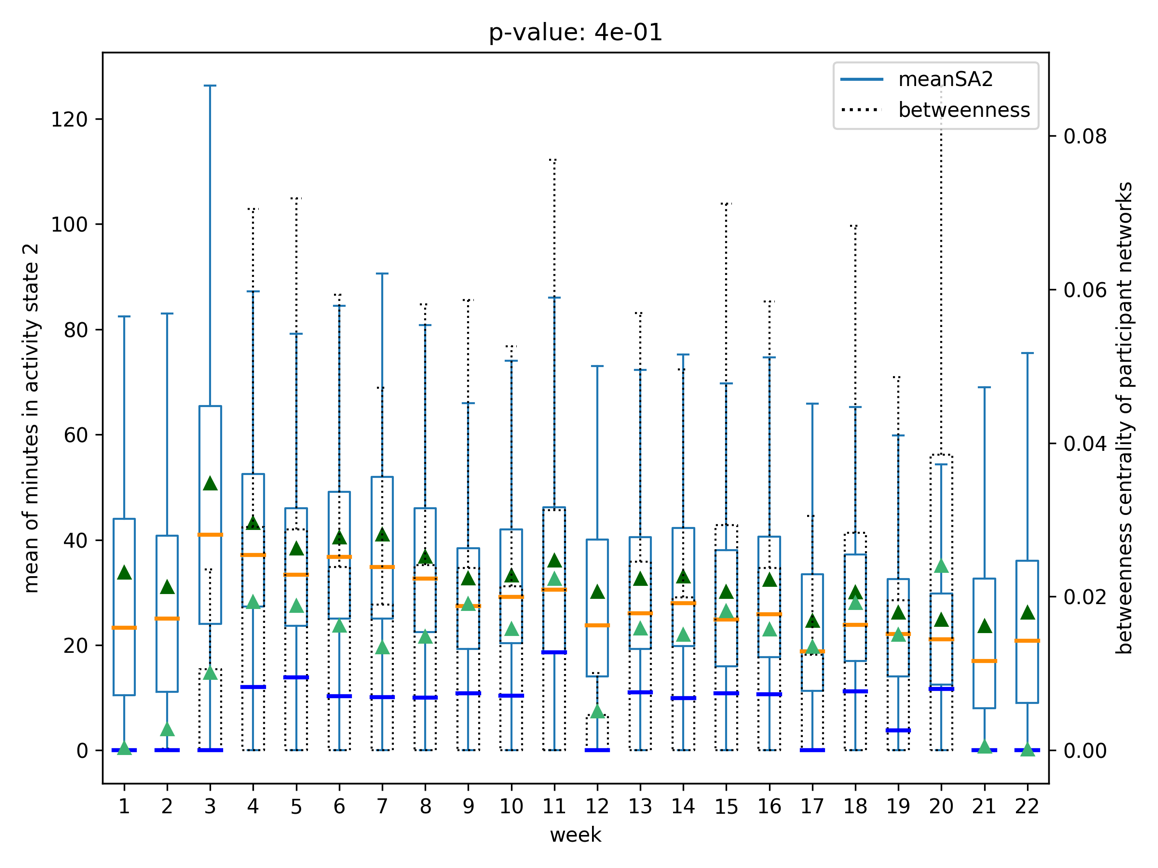


Figure 40 box plot of mean of minutes in state 2 and betweenness centrality of participant network


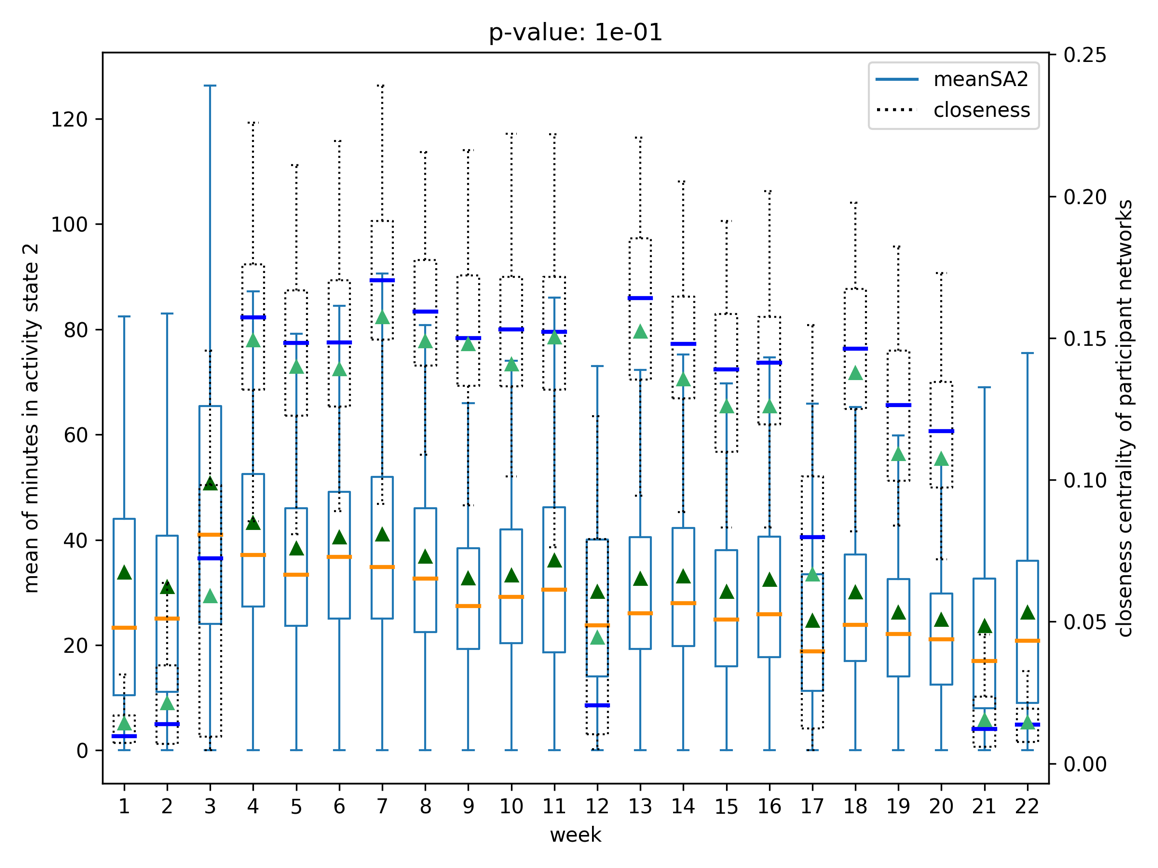


Figure 41 box plot of mean of minutes in state 2 and closeness centrality of participant network


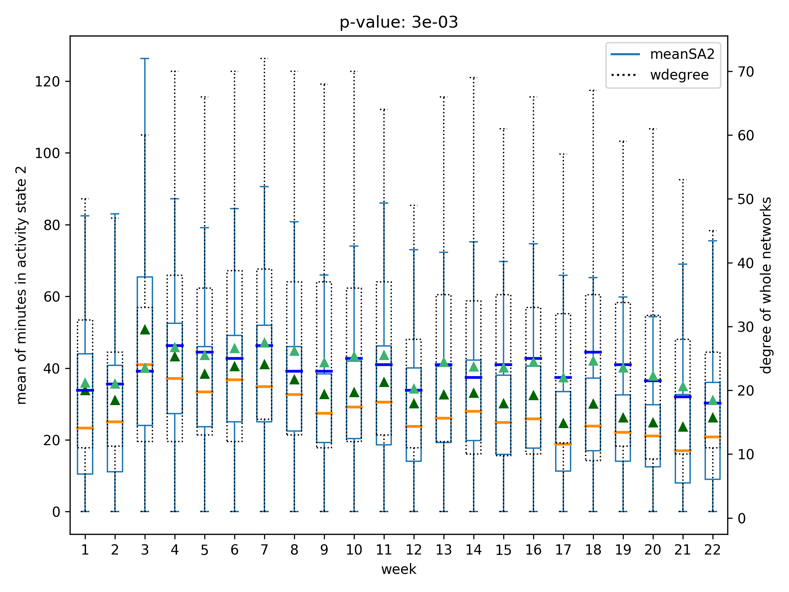


Figure 42 box plot of mean of minutes in state 2 and degree of whole network


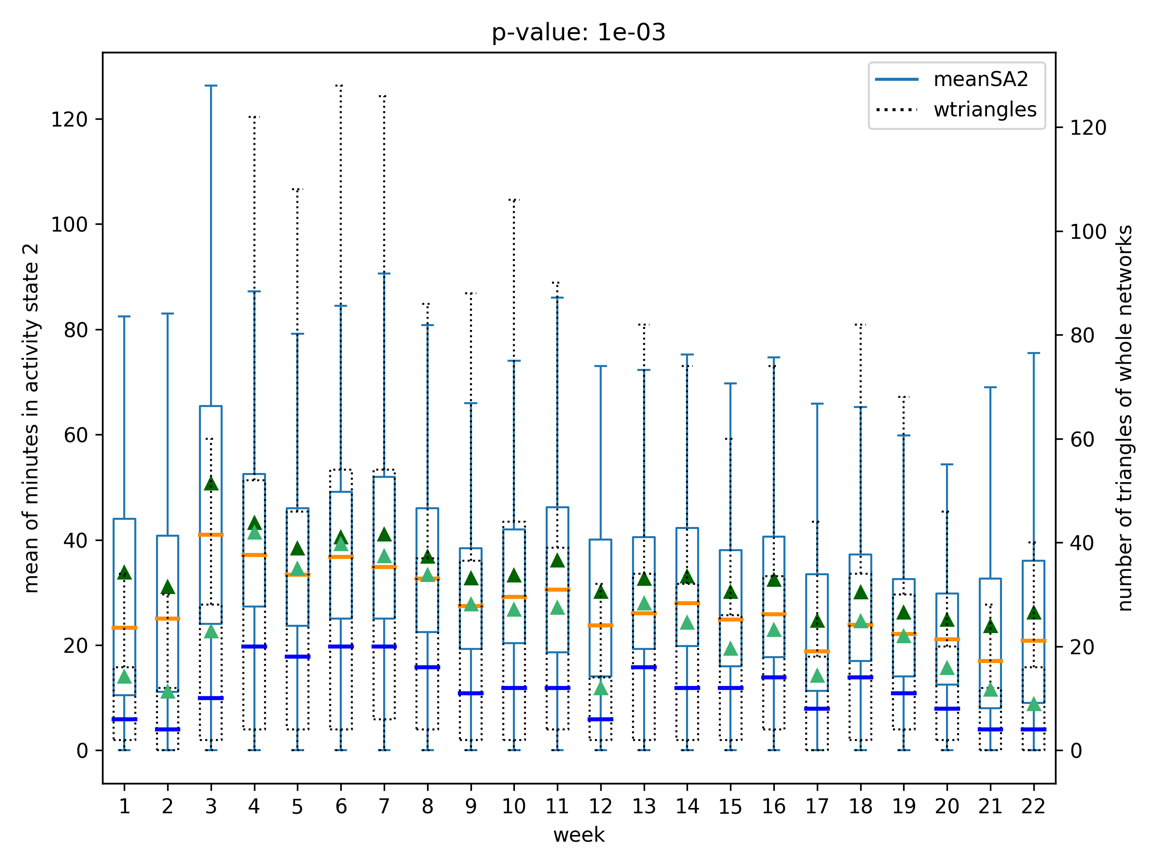


Figure 43 box plot of mean of minutes in state 2 and number of triangles of whole network


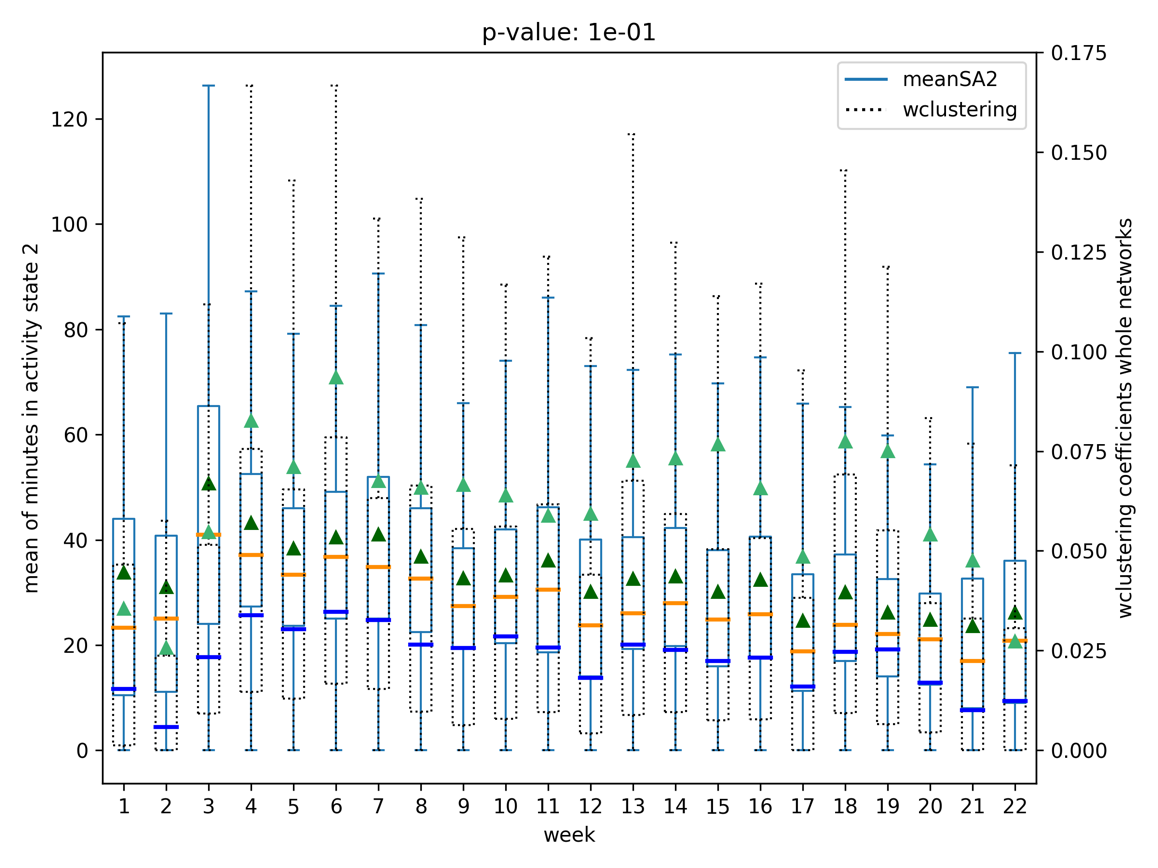


Figure 44 box plot of mean of minutes in state 2 and clustering coefficients of whole network


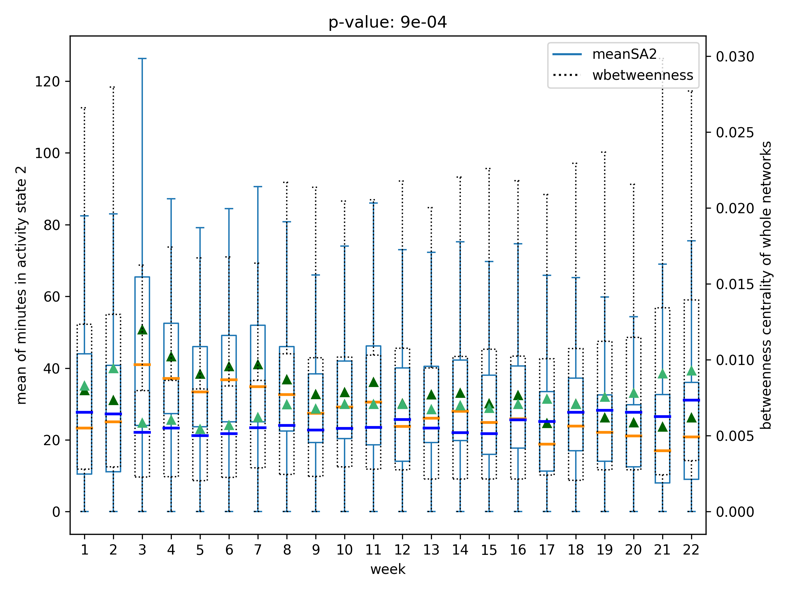


Figure 45 box plot of mean of minutes in state 2 and betweenness centrality of whole network


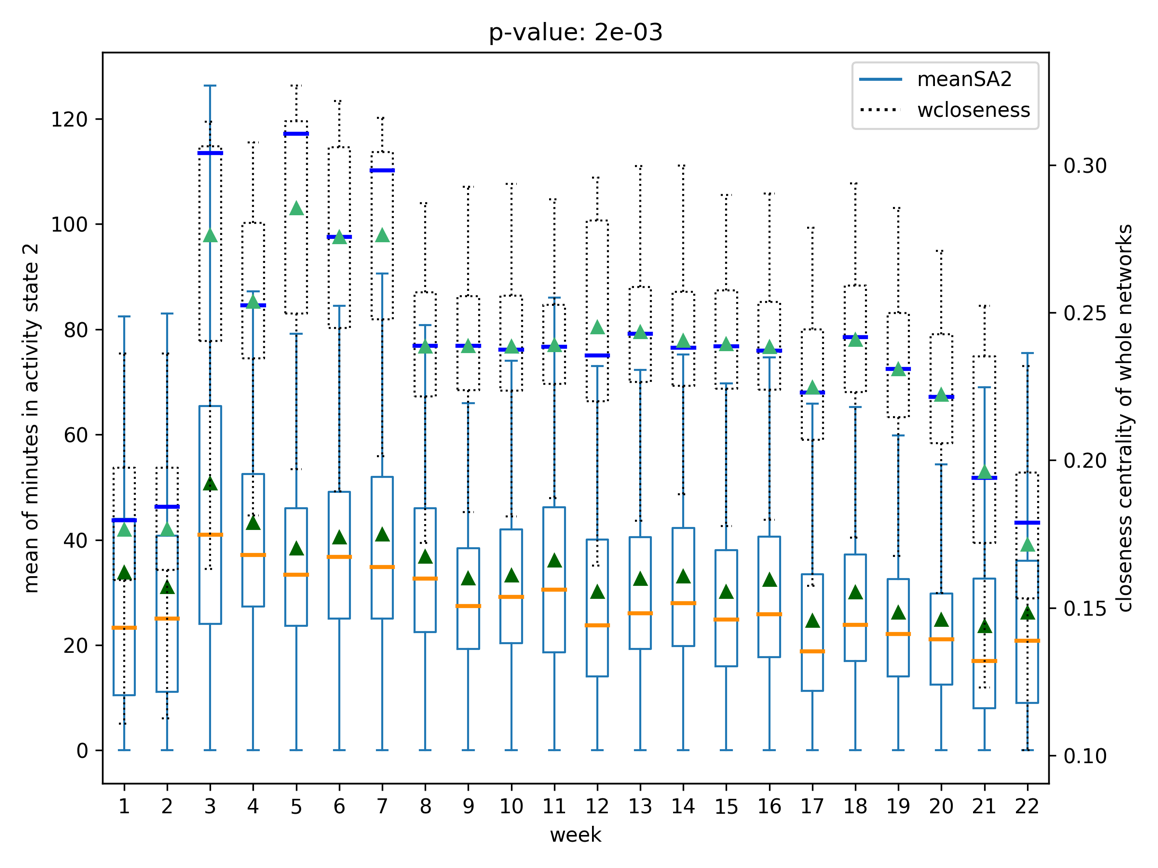


Figure 46 box plot of mean of minutes in state 2 and closeness centrality of whole network


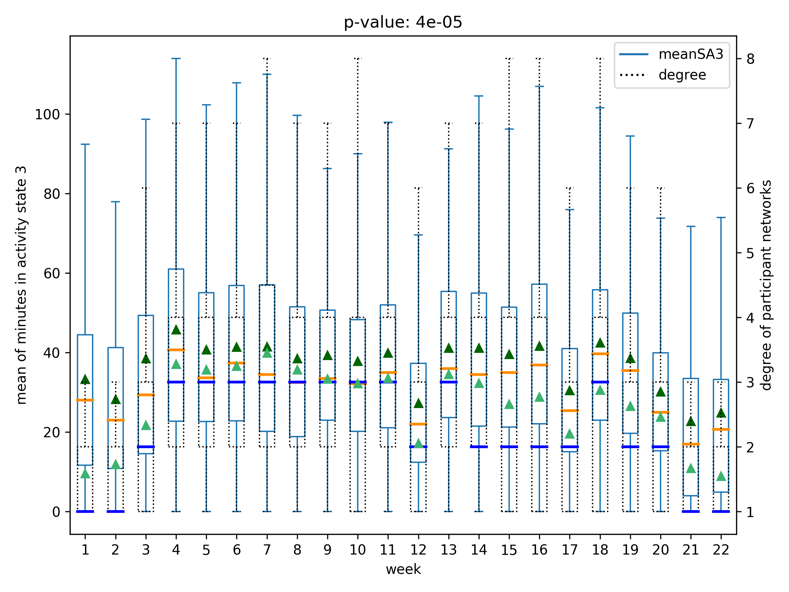


Figure 47 box plot of mean of minutes in state 3 and degree of participant network


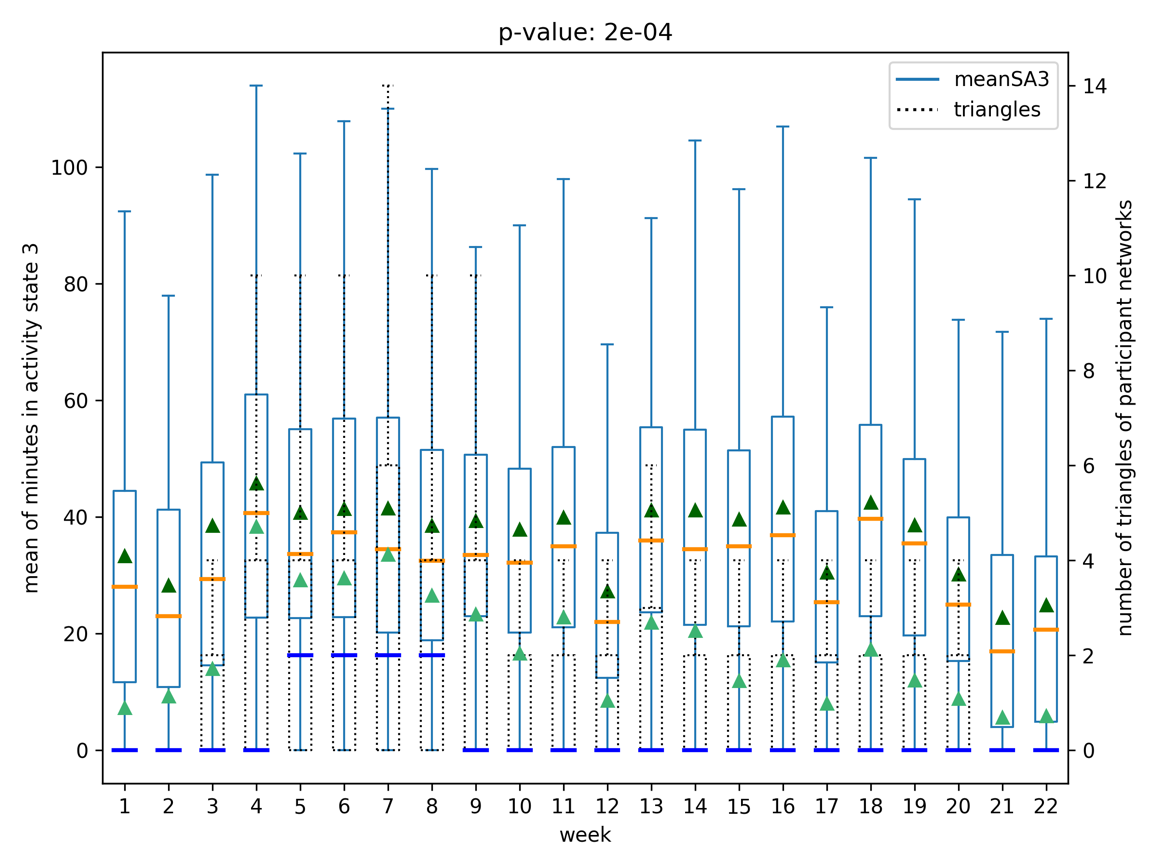


Figure 48 box plot of mean of minutes in state 3 and number of triangles of participant network


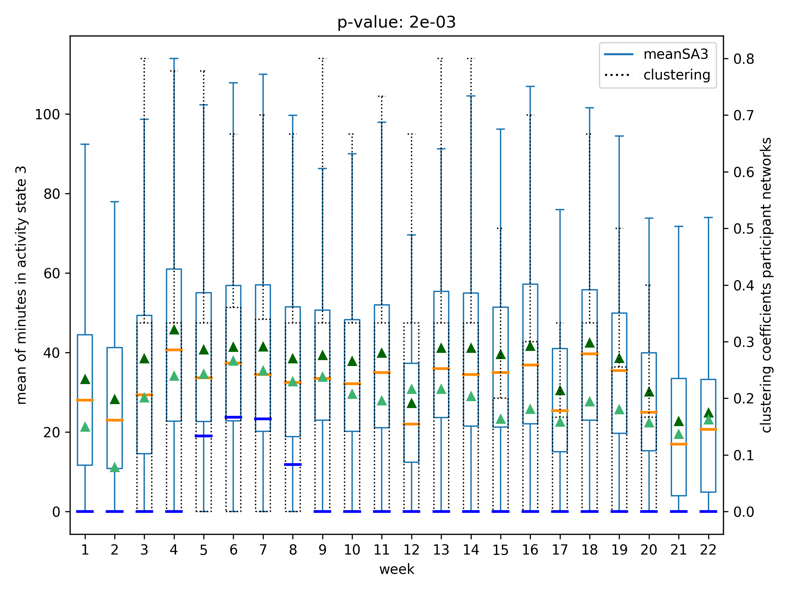


Figure 49 box plot of mean of minutes in state 3 and clustering coefficients of participant network


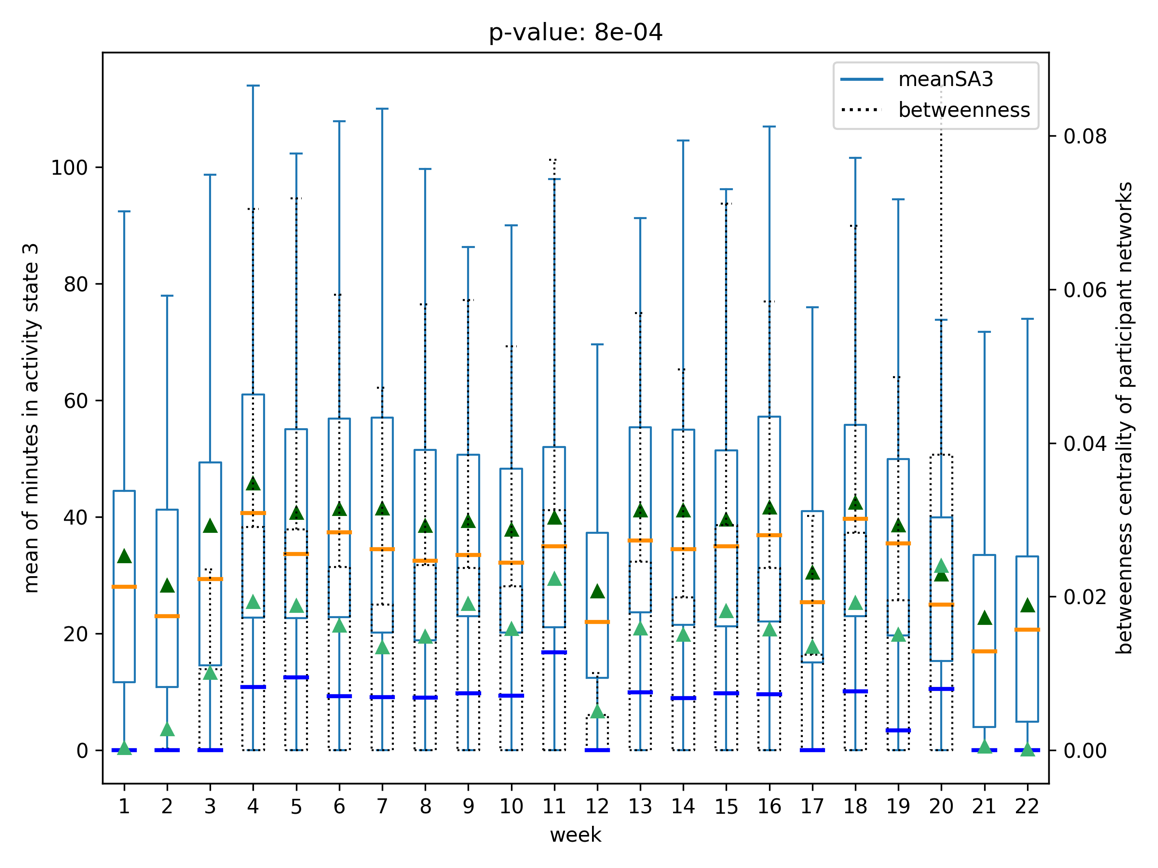


Figure 50 box plot of mean of minutes in state 3 and betweenness centrality of participant network


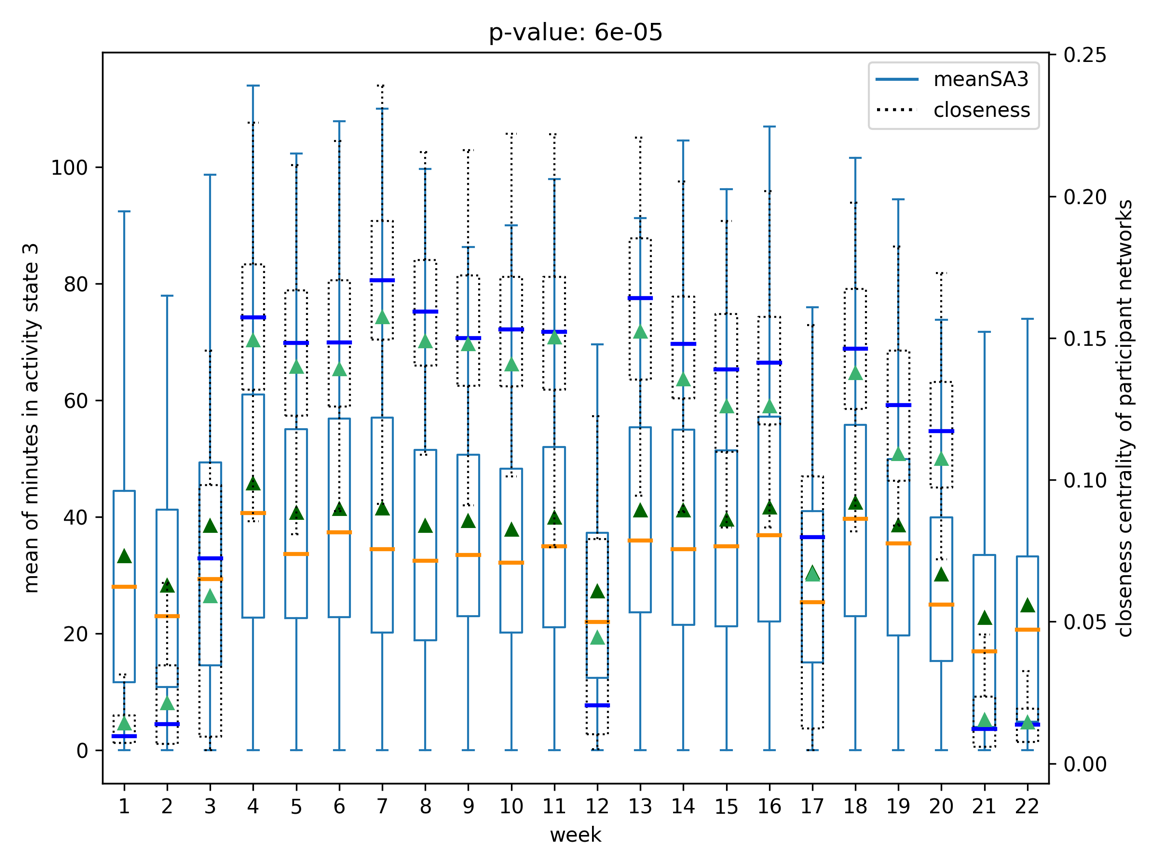


Figure 51 box plot of mean of minutes in state 3 and closeness centrality of participant network


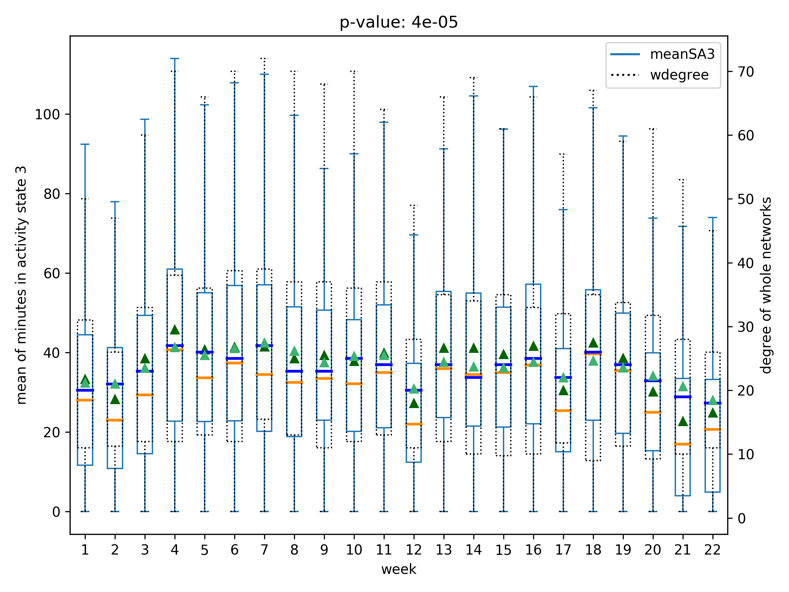


Figure 52 box plot of mean of minutes in state 3 and degree of whole network


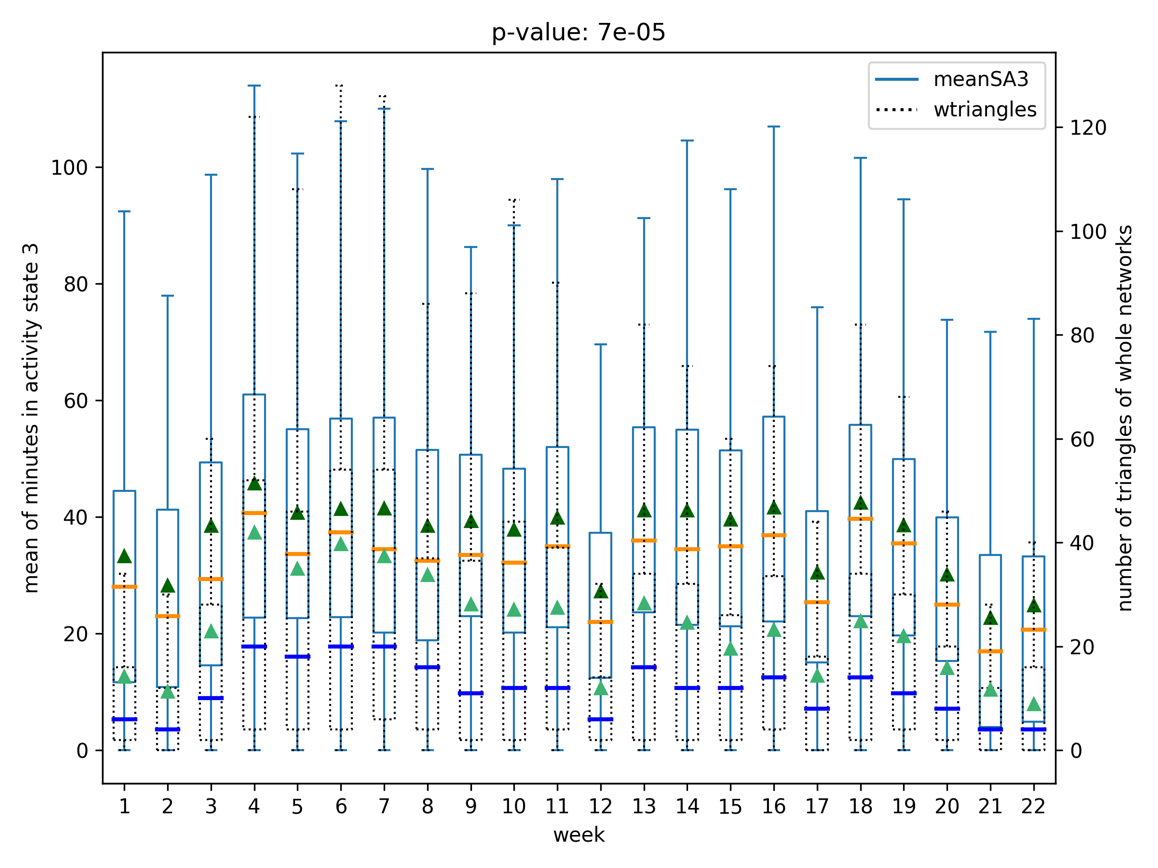


Figure 53 box plot of mean of minutes in state 3 and number of triangles of whole network


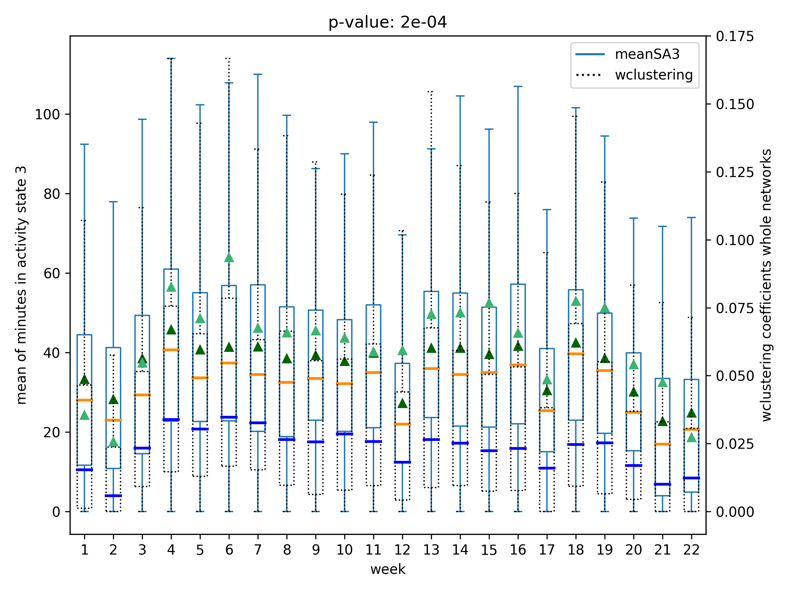


Figure 54 box plot of mean of minutes in state 3 and clustering coefficients of whole network


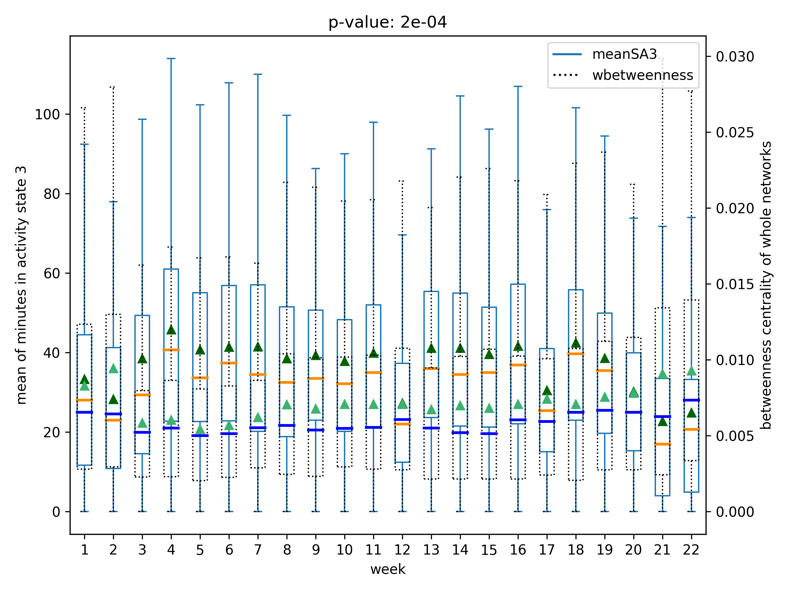


Figure 55 box plot of mean of minutes in state 3 and betweenness centrality of whole network


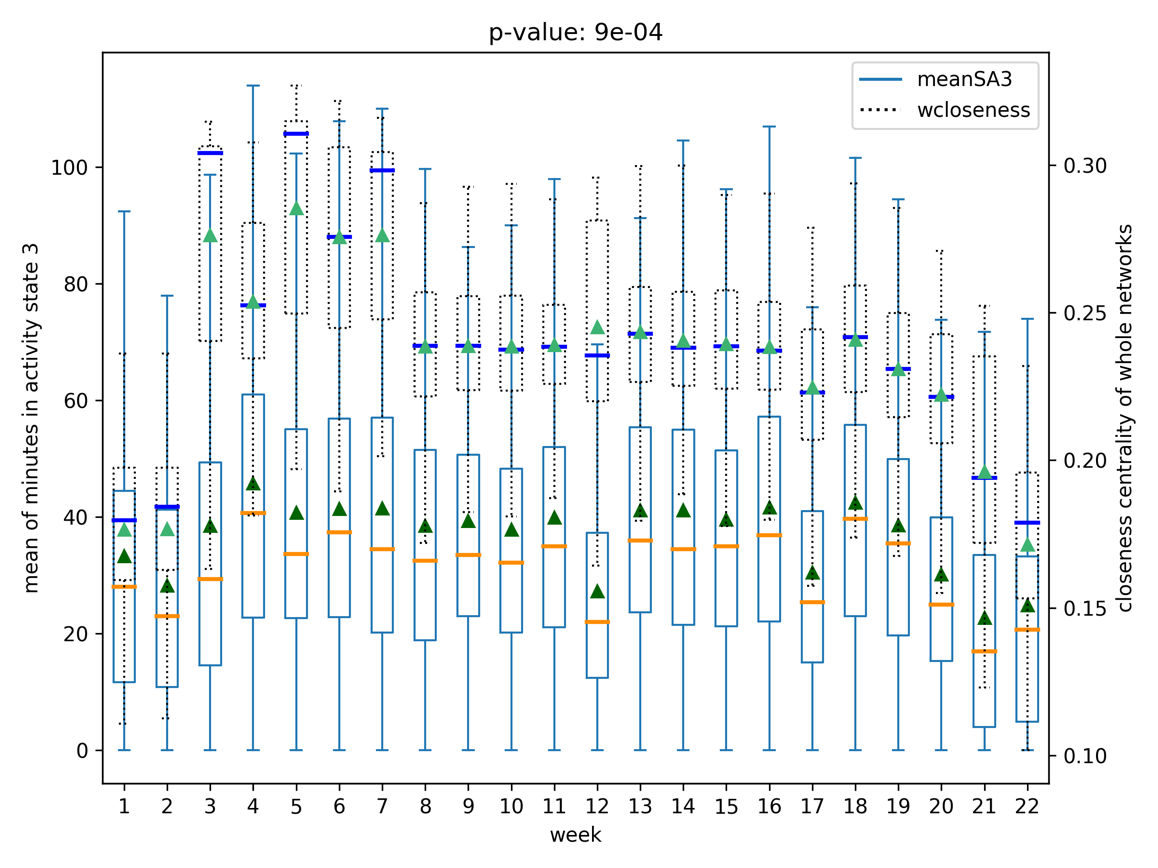


Figure 56 box plot of mean of minutes in state 3 and closeness centrality of whole network
